# Supplementary material for: Incorporating variation in death times improves predictions of ectotherm responses to stressful temperatures
Source: PLoS Biol. 2026 May 21;24(5):e3003623. doi: 10.1371/journal.pbio.3003623 (PMC13221141; doi:10.1371/journal.pbio.3003623)
Supplement: S1 Table — Models were fit by regressing time to failure on temperature, allowing the location parameter to vary with temperature and shape to remain constant, with the exception of the Exponential distribution, which has no shape parameter. The data underlying this Figure can be found in https://zenodo.org/records/1937403. (PDF) [file pbio.3003623.s001.pdf]

S1 Table. AIC values for each of five commonly used parametric survival models for static temperature TDT data from 11 species of *Drosophila*. Models were fit by regressing time to failure on temperature, allowing the location parameter to vary with temperature and shape to remain constant, with the exception of the Exponential distribution, which has no shape parameter. The data underlying this Figure can be found in https://zenodo.org/records/1937403.

	Exponential	Weibull	Log-Logistic	Log-Normal	Gompertz
D. buzzatii	1386.7161	1133.3702	1110.0484	1115.4445	1369.7699
D. equinoxialis	1088.3253	924.3640	900.8418	901.5934	1061.7020
D. immigrans	1205.5525	1019.9791	1018.0462	1028.7044	1147.6177
D. melanogaster	1336.3529	1178.6644	1160.2882	1163.2129	1264.0495
D. mercatorum	1255.4791	1106.7456	1094.6618	1088.7687	1151.6566
D. mojavensis	1283.3209	1150.6634	1118.7615	1123.2064	1237.0203
D. montana	982.8214	837.3137	832.0108	837.0622	920.8424
D. rufa	822.0971	739.8311	714.0270	718.9075	772.8995
D. subobscura	811.0802	725.8633	730.5606	731.3250	764.1026
D. suzukii	1104.7212	962.0355	945.1548	952.8447	1044.9274
D. virilis	883.6984	798.9874	791.1950	796.8057	851.4679
