# Supplementary material for: Incorporating variation in death times improves predictions of ectotherm responses to stressful temperatures
Source: PLoS Biol. 2026 May 21;24(5):e3003623. doi: 10.1371/journal.pbio.3003623 (PMC13221141; doi:10.1371/journal.pbio.3003623)
Supplement: S2 Table — Models were fit by regressing time to failure on temperature, allowing the location parameter to vary with temperature and shape to remain constant, with the exception of the Exponential distribution, which has no shape parameter. The Log-Logistic model had the best fit 9 out of 11 times, while the Exponential distribution is the worst fit every time. The data underlying this Figure can be found in https://zenodo.org/records/1937403. (PDF) [file pbio.3003623.s002.pdf]

S2 Table. Parametric survival models ranked from lowest AIC value (1) to highest AIC value (5) based on the fits reported in Table A. Models were fit by regressing time to failure on temperature, allowing the location parameter to vary with temperature and shape to remain constant, with the exception of the Exponential distribution, which has no shape parameter. The Log-Logistic model had the best fit 9 out of 11 times, while the Exponential distribution is the worst fit every time. The data underlying this Figure can be found in https://zenodo.org/records/1937403.

	Exponential	Weibull	Log-Logistic	Log-Normal	Gompertz
D. buzzatii	5	3	1	2	4
D. equinoxialis	5	3	1	2	4
D. immigrans	5	2	1	3	4
D. melanogaster	5	3	1	2	4
D. mercatorum	5	3	2	1	4
D. mojavensis	5	3	1	2	4
D. montana	5	3	1	2	4
D. rufa	5	3	1	2	4
D. subobscura	5	1	2	3	4
D. suzukii	5	3	1	2	4
D. virilis	5	3	1	2	4
