# Supplementary material for: Incorporating variation in death times improves predictions of ectotherm responses to stressful temperatures
Source: PLoS Biol. 2026 May 21;24(5):e3003623. doi: 10.1371/journal.pbio.3003623 (PMC13221141; doi:10.1371/journal.pbio.3003623)
Supplement: S3 Table — The data underlying this Figure can be found in https://zenodo.org/records/1937403. (PDF) [file pbio.3003623.s003.pdf]

S3 Table. Sample sizes trials by combination of species and temperature after filtering for only trials with 10 or more data points. The data underlying this Figure can be found in https://zenodo.org/records/1937403.

	D. buzzatii	D. equinoxialis	D. immigrans	D. melanogaster	D. mercatorum
33	0	0	0	0	0
33.5	0	0	10	0	0
34	0	13	0	0	0
34.5	0	0	10	0	0
35	0	0	10	0	0
35.5	0	10	10	0	10
36	0	12	10	11	10
36.5	0	10	10	10	10
37	0	0	17	15	10
37.5	0	13	10	10	10
38	0	10	16	14	0
38.5	10	10	10	10	10
39	10	0	0	10	10
39.5	10	10	0	10	10
40	10	13	0	10	10
40.5	10	0	0	10	10
41	10	0	0	10	0
41.5	25	0	0	0	0
42	0	0	0	0	0
42.5	20	0	0	0	0
43	10	0	0	0	0
43.5	0	0	0	0	0

	D. mojavensis	D. montana	D. rufa	D. subobscura	D. suzukii	D. virilis
33	0	0	0	10	0	0
33.5	0	0	0	10	0	0
34	0	0	10	11	0	0
34.5	0	0	10	10	0	0
35	0	0	10	10	10	0
35.5	0	0	10	10	10	0
36	0	10	10	10	10	0
36.5	0	10	10	10	10	0
37	0	12	10	12	26	0
37.5	0	0	10	10	10	10
38	0	0	15	10	20	10
38.5	0	13	10	10	0	0
39	0	10	10	0	10	11
39.5	10	13	10	10	0	10
40	10	0	0	10	0	10
40.5	0	0	0	0	0	10
41	13	0	0	0	0	0
41.5	18	0	0	0	0	10
42	10	0	0	0	0	0
42.5	10	0	0	0	0	0
43	10	0	0	0	0	0
43.5	10	0	0	0	0	0
