# Supplementary material for: Incorporating variation in death times improves predictions of ectotherm responses to stressful temperatures
Source: PLoS Biol. 2026 May 21;24(5):e3003623. doi: 10.1371/journal.pbio.3003623 (PMC13221141; doi:10.1371/journal.pbio.3003623)

S1 Figure. This figure illustrates how failure density, cumulative survival, and hazard at some constant temperature vary with the *shape* parameters. Line color indicates *shape* (0.5, 1, 2, 3, 4). Note how *shape* influences how hazard increases with time, variation in failure times, and the curvature of cumulative survival over time. The data underlying this Figure can be found in https://zenodo.org/records/1937403.

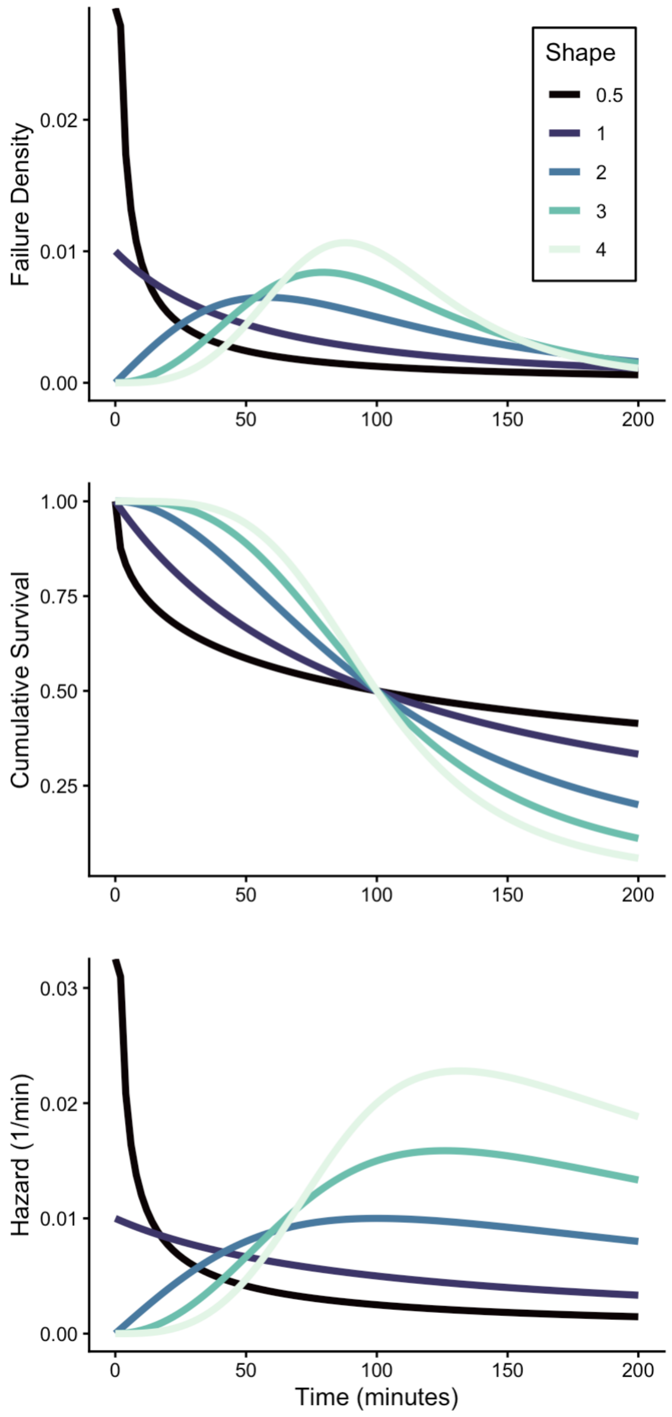

Supplement: S1 Fig — Line color indicates shape (0.5, 1, 2, 3, 4). Note how shape influences how hazard increases with time, variation in failure times, and the curvature of cumulative survival over time. The data underlying this Figure can be found in https://zenodo.org/records/1937403. (PDF) [file pbio.3003623.s004.pdf]
