# Supplementary material for: Incorporating variation in death times improves predictions of ectotherm responses to stressful temperatures
Source: PLoS Biol. 2026 May 21;24(5):e3003623. doi: 10.1371/journal.pbio.3003623 (PMC13221141; doi:10.1371/journal.pbio.3003623)

S2 Figure. Residual plots for failure time (t_f) as a function of temperature for 11 *Drosophila* species. Based on data from Jørgensen *et al* (2019) for knockdown times for adult *Drosophila*. Residuals do not vary consistently with temperature across species, indicating that the TDT assumption of a linear relationship between log(t_f) and temperature holds for these datasets. The data underlying this Figure can be found in https://zenodo.org/records/1937403.

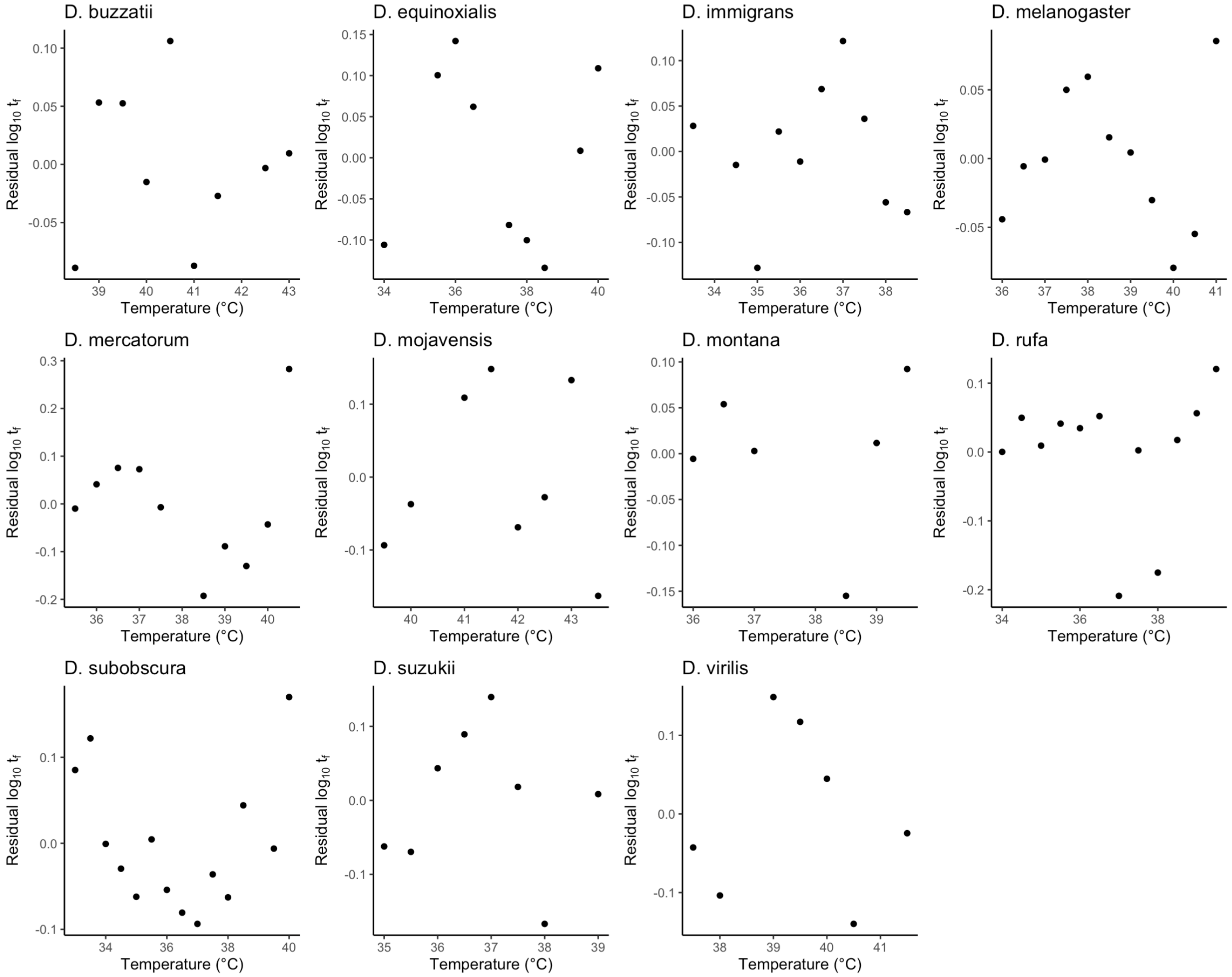

Supplement: S2 Fig — Based on data from Jørgensen and colleagues (2019) for knockdown times for adult Drosophila. Residuals do not vary consistently with temperature across species, indicating that the TDT assumption of a linear relationship between log(tf) and temperature holds for these datasets. The data underlying this Figure can be found in https://zenodo.org/records/1937403. (PDF) [file pbio.3003623.s005.pdf]
