# Supplementary material for: Incorporating variation in death times improves predictions of ectotherm responses to stressful temperatures
Source: PLoS Biol. 2026 May 21;24(5):e3003623. doi: 10.1371/journal.pbio.3003623 (PMC13221141; doi:10.1371/journal.pbio.3003623)
Supplement: S3 Fig — Data from Fig 2 expressed for each species separately for clarity. The data underlying this Figure can be found in https://zenodo.org/records/1937403. (PDF) [file pbio.3003623.s006.pdf]

S3 Figure. Changes in median (A) and (C) variance in adult failure (knockdown) times t_f, and in estimated log-logistic *scale* (B) and *shape* (D) parameters, as functions of temperature for 11 *Drosophila* species. Data from Figure 2 expressed for each species separately for clarity. The data underlying this Figure can be found in https://zenodo.org/records/1937403.

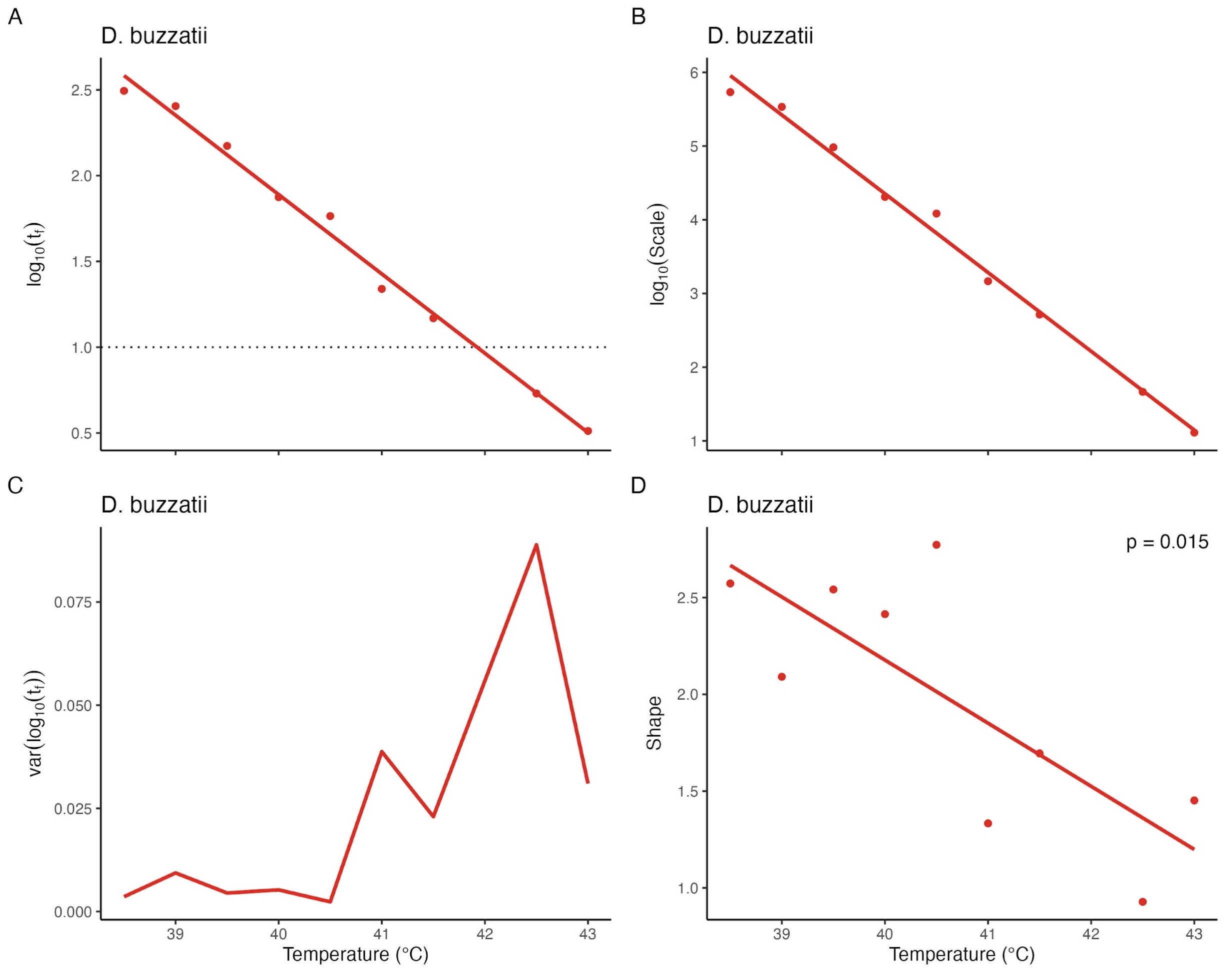

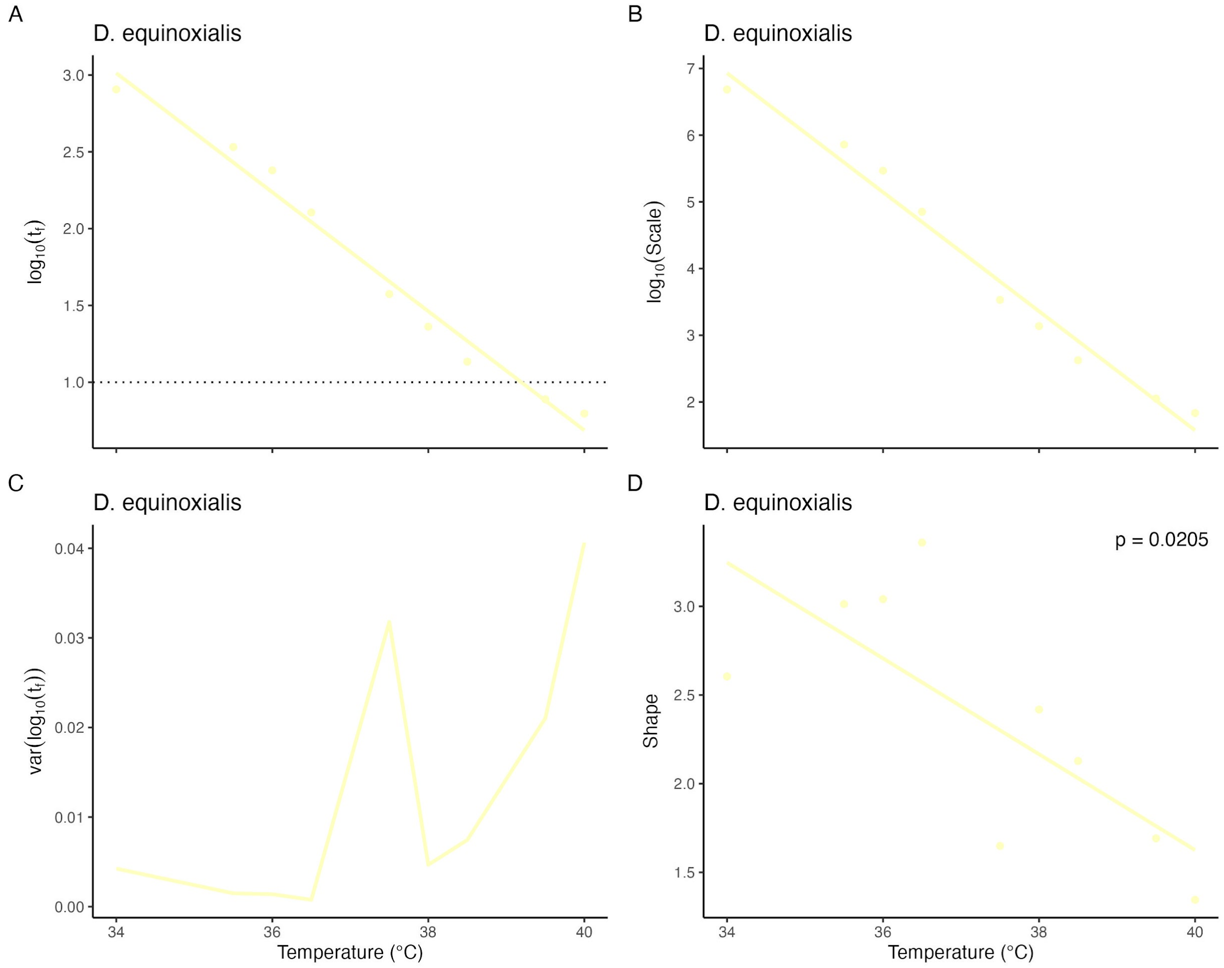
A
D. equinoxialis
3.0
2.5
2.0
1.5
1.0
$log_{10}(t_f)$
B
D. equinoxialis
7
6
5
4
3
2
log_{10}(Scale)
C
D. equinoxialis
0.04
0.03
0.02
0.01
0.00
var($log_{10}(t_f)$)
34
36
38
40
Temperature (°C)
D
D. equinoxialis
p = 0.0205
3.0
2.5
2.0
1.5
Shape
34
36
38
40
Temperature (°C)

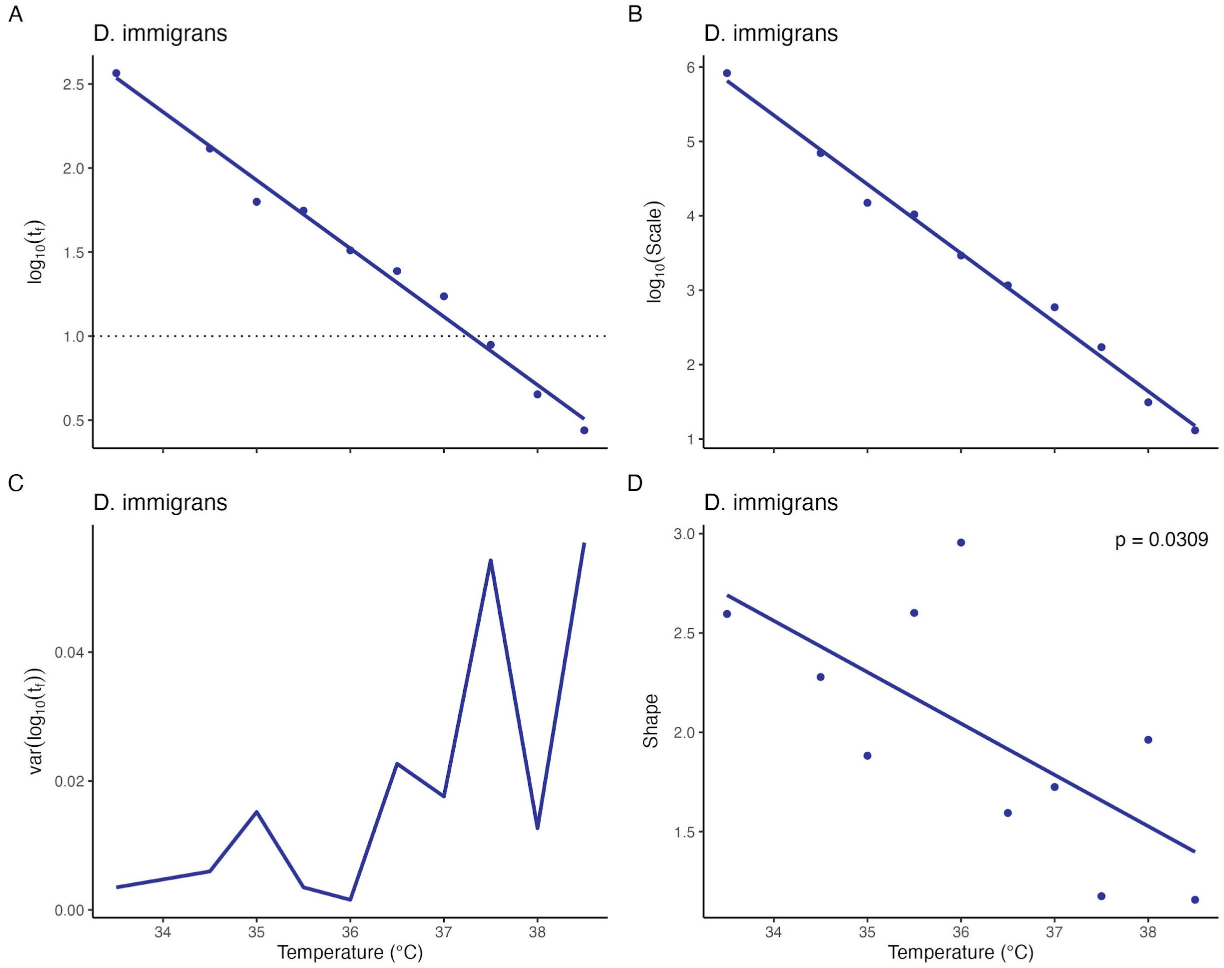
A
D. immigrans
$\log_{10}(t_f)$
2.5
2.0
1.5
1.0
0.5
B
D. immigrans
$\log_{10}(\text{Scale})$
6
5
4
3
2
1
C
D. immigrans
$\text{var}(\log_{10}(t_f))$
0.04
0.02
0.00
34
35
36
37
38
Temperature (°C)
D
D. immigrans
p = 0.0309
Shape
3.0
2.5
2.0
1.5
34
35
36
37
38
Temperature (°C)

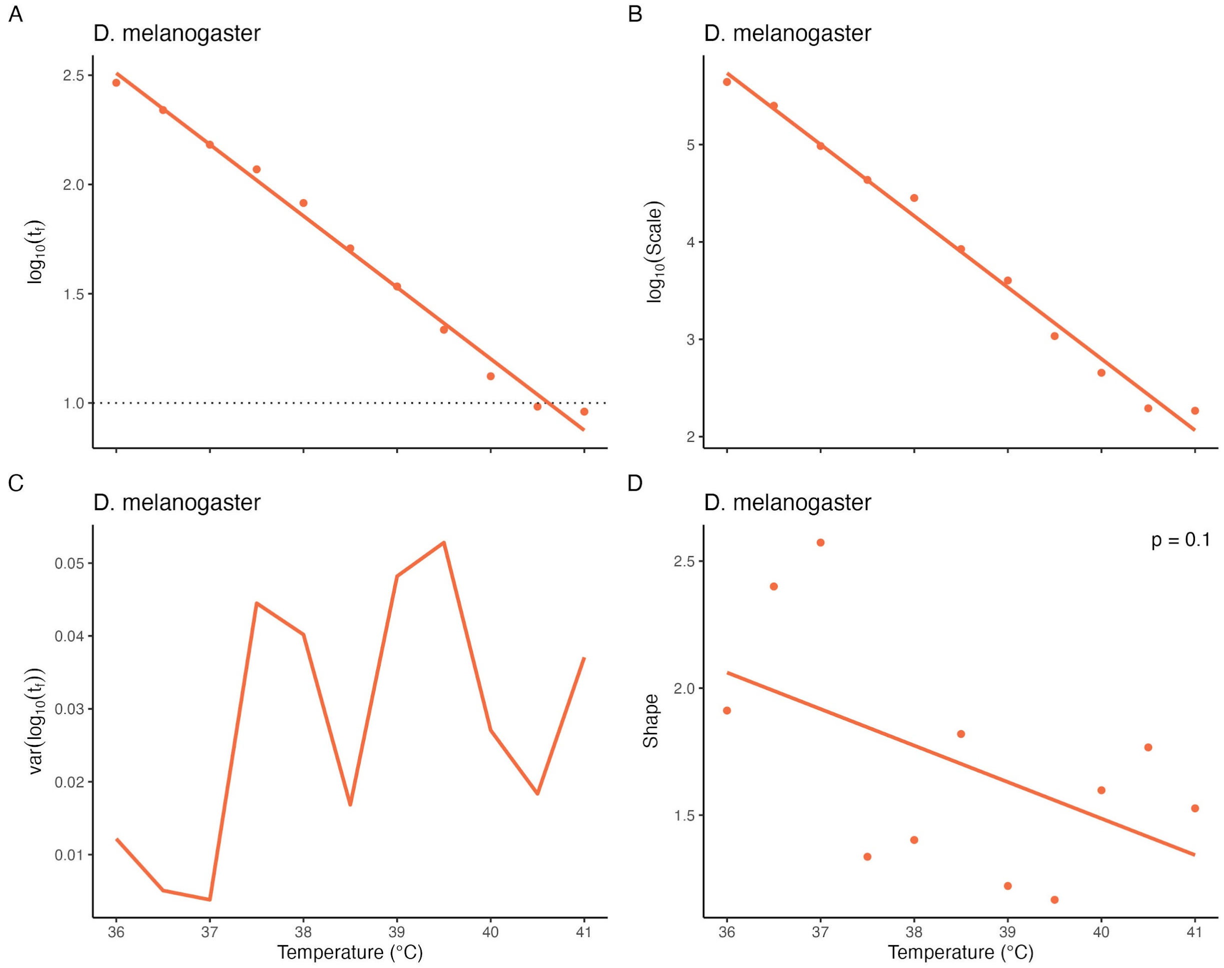
A
D. melanogaster
log10(tf)
2.5
2.0
1.5
1.0
B
D. melanogaster
log10(Scale)
5
4
3
2
C
D. melanogaster
var(log10(tf))
0.05
0.04
0.03
0.02
0.01
36
37
38
39
40
41
Temperature (°C)
D
D. melanogaster
p = 0.1
Shape
2.5
2.0
1.5
36
37
38
39
40
41
Temperature (°C)

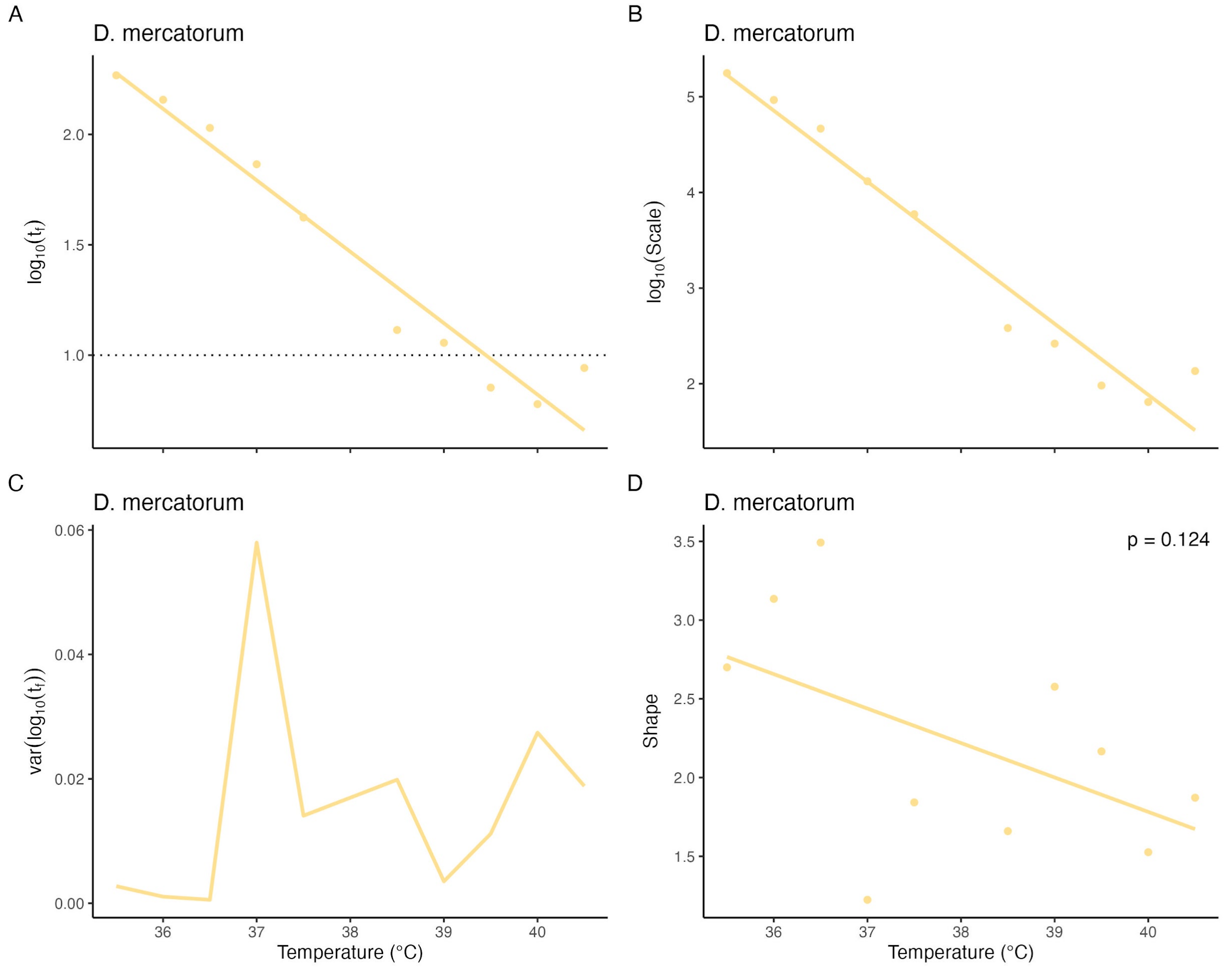
A
D. mercatorum
log10(tf)
2.0
1.5
1.0
B
D. mercatorum
log10(Scale)
5
4
3
2
C
D. mercatorum
var(log10(tf))
0.06
0.04
0.02
0.00
36
37
38
39
40
Temperature (°C)
D
D. mercatorum
p = 0.124
Shape
3.5
3.0
2.5
2.0
1.5
36
37
38
39
40
Temperature (°C)

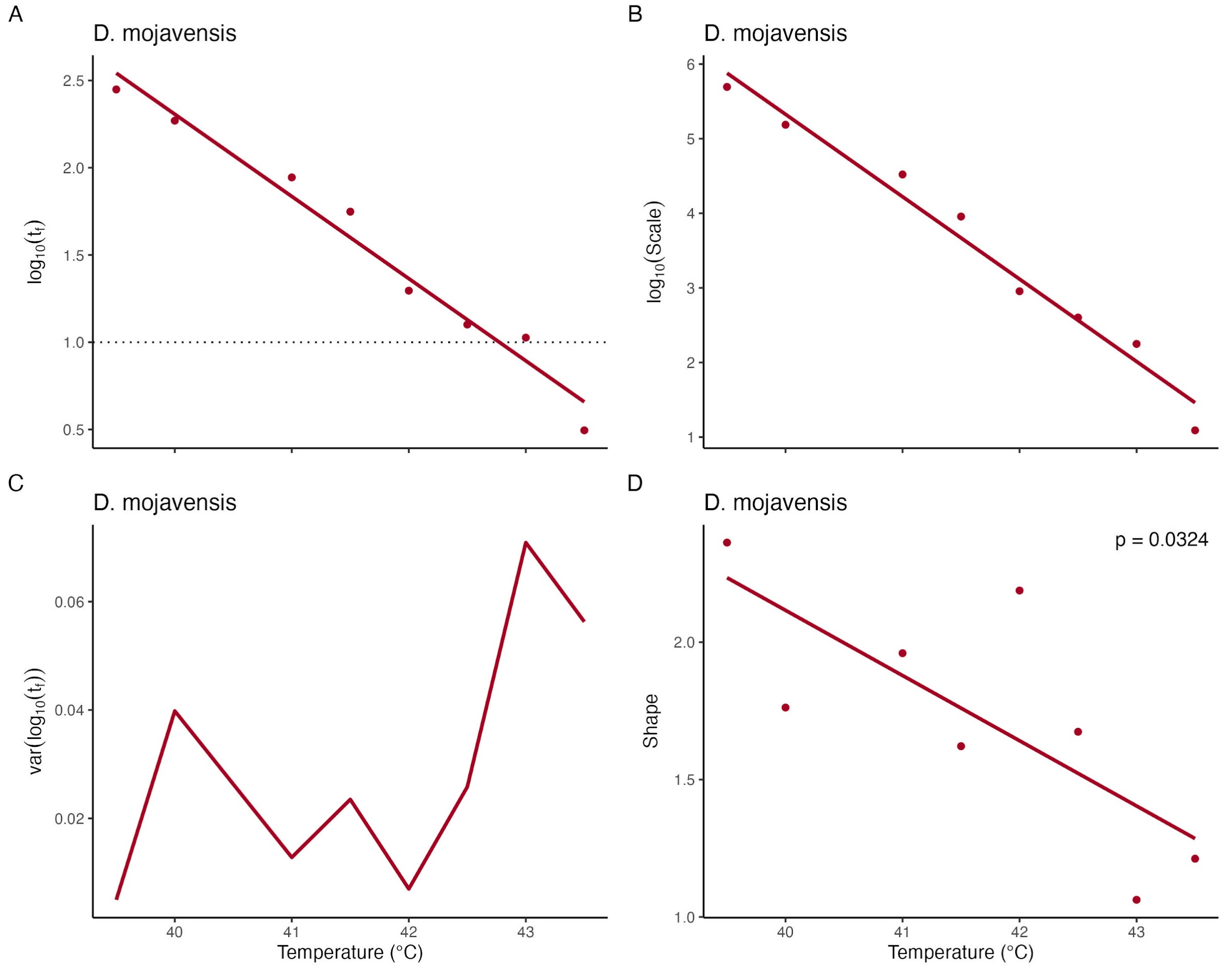
A
D. mojavensis
log10(tf)
2.5
2.0
1.5
1.0
0.5
B
D. mojavensis
log10(Scale)
6
5
4
3
2
1
C
D. mojavensis
var(log10(tf))
0.06
0.04
0.02
40
41
42
43
Temperature (°C)
D
D. mojavensis
p = 0.0324
Shape
2.0
1.5
1.0
40
41
42
43
Temperature (°C)

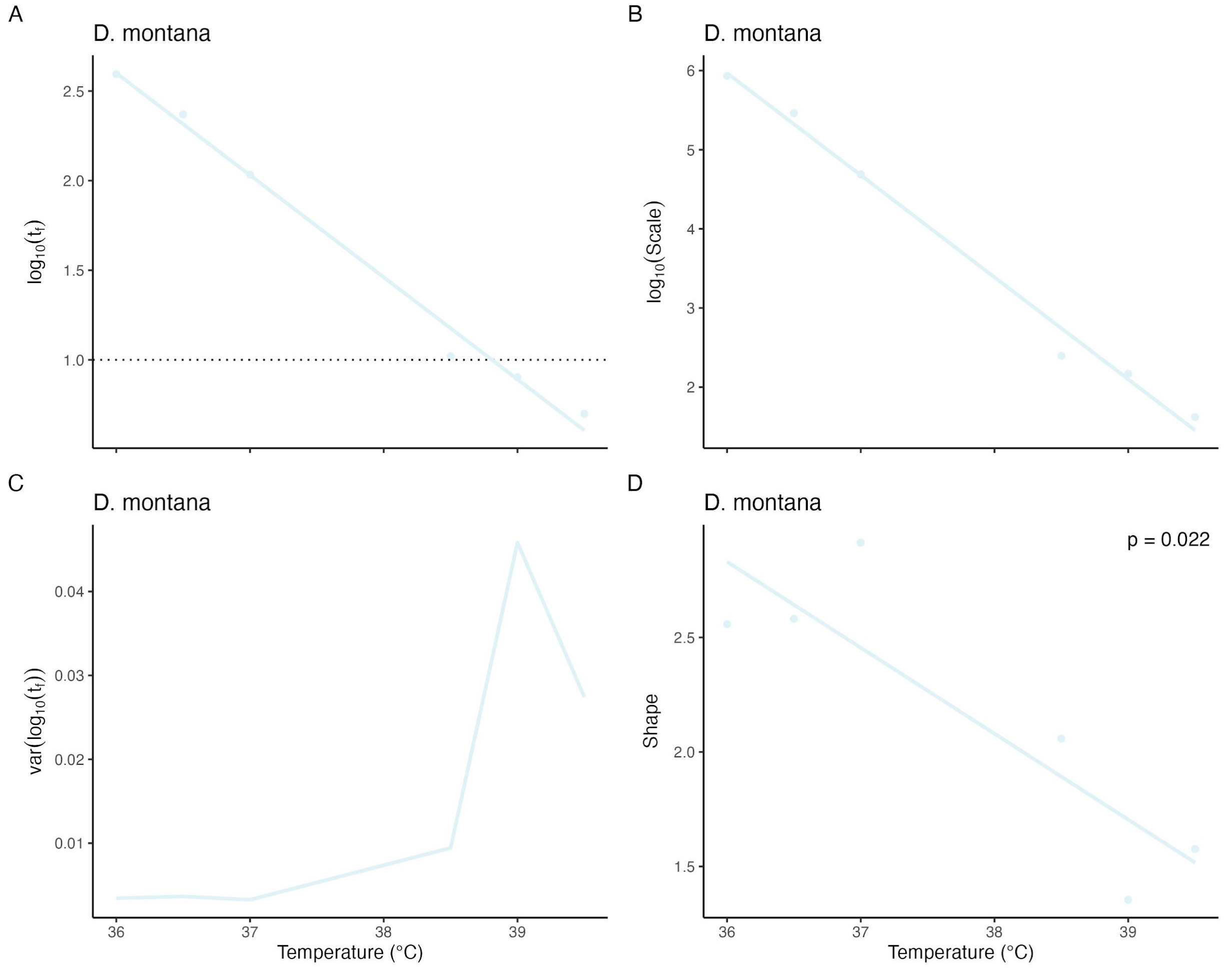
A
D. montana
log10(tf)
2.5
2.0
1.5
1.0
B
D. montana
log10(Scale)
6
5
4
3
2
C
D. montana
var(log10(tf))
0.04
0.03
0.02
0.01
36
37
38
39
Temperature (°C)
D
D. montana
p = 0.022
Shape
2.5
2.0
1.5
36
37
38
39
Temperature (°C)

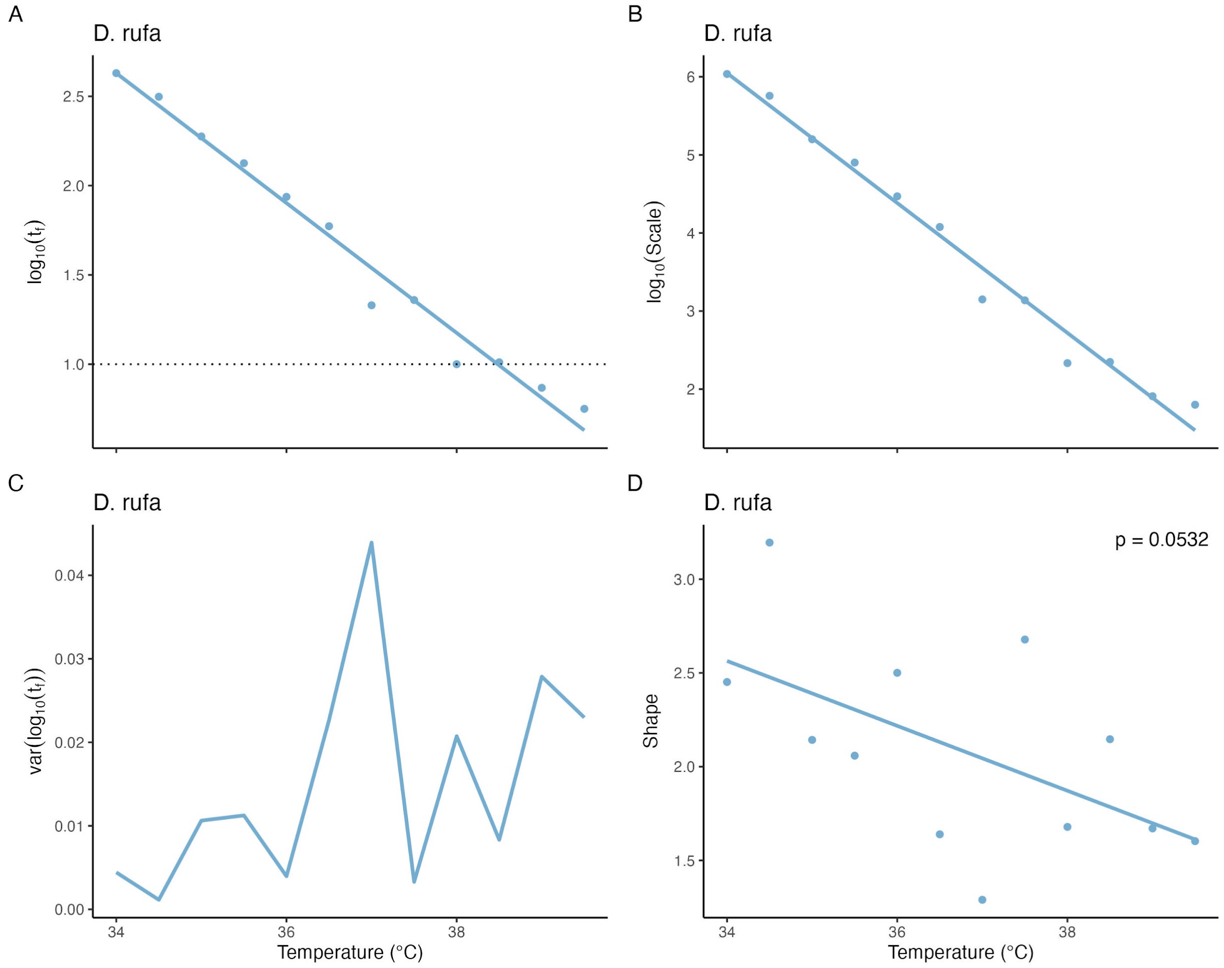
A
D. rufa
log10(tf)
2.5
2.0
1.5
1.0
B
D. rufa
log10(Scale)
6
5
4
3
2
C
D. rufa
var(log10(tf))
0.04
0.03
0.02
0.01
0.00
34
36
38
Temperature (°C)
D
D. rufa
p = 0.0532
Shape
3.0
2.5
2.0
1.5
34
36
38
Temperature (°C)

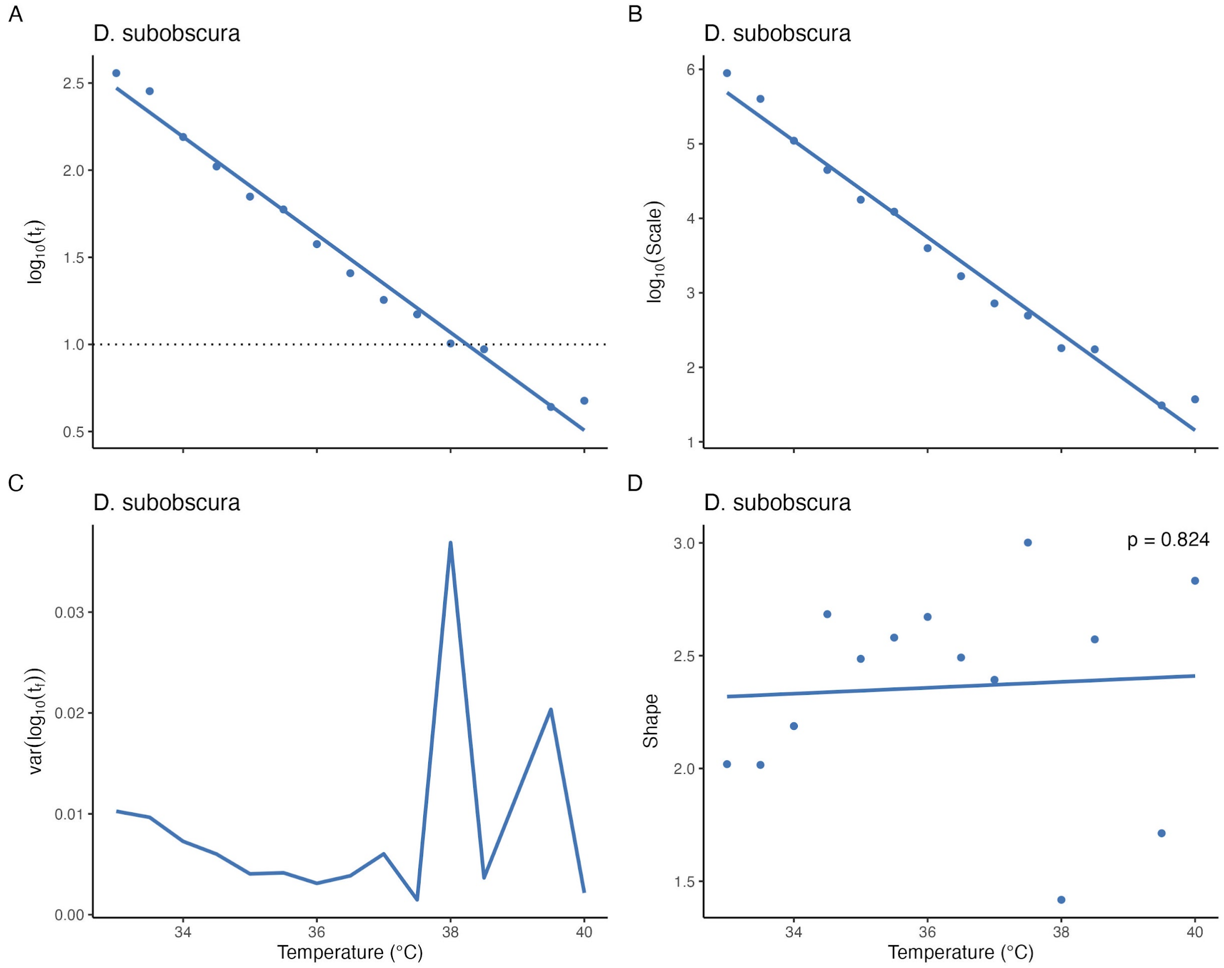
A
D. subobscura
$log_{10}(t_f)$
2.5
2.0
1.5
1.0
0.5
B
D. subobscura
$log_{10}(Scale)$
6
5
4
3
2
1
C
D. subobscura
$var(log_{10}(t_f))$
0.03
0.02
0.01
0.00
34
36
38
40
Temperature (°C)
D
D. subobscura
p = 0.824
Shape
3.0
2.5
2.0
1.5
34
36
38
40
Temperature (°C)

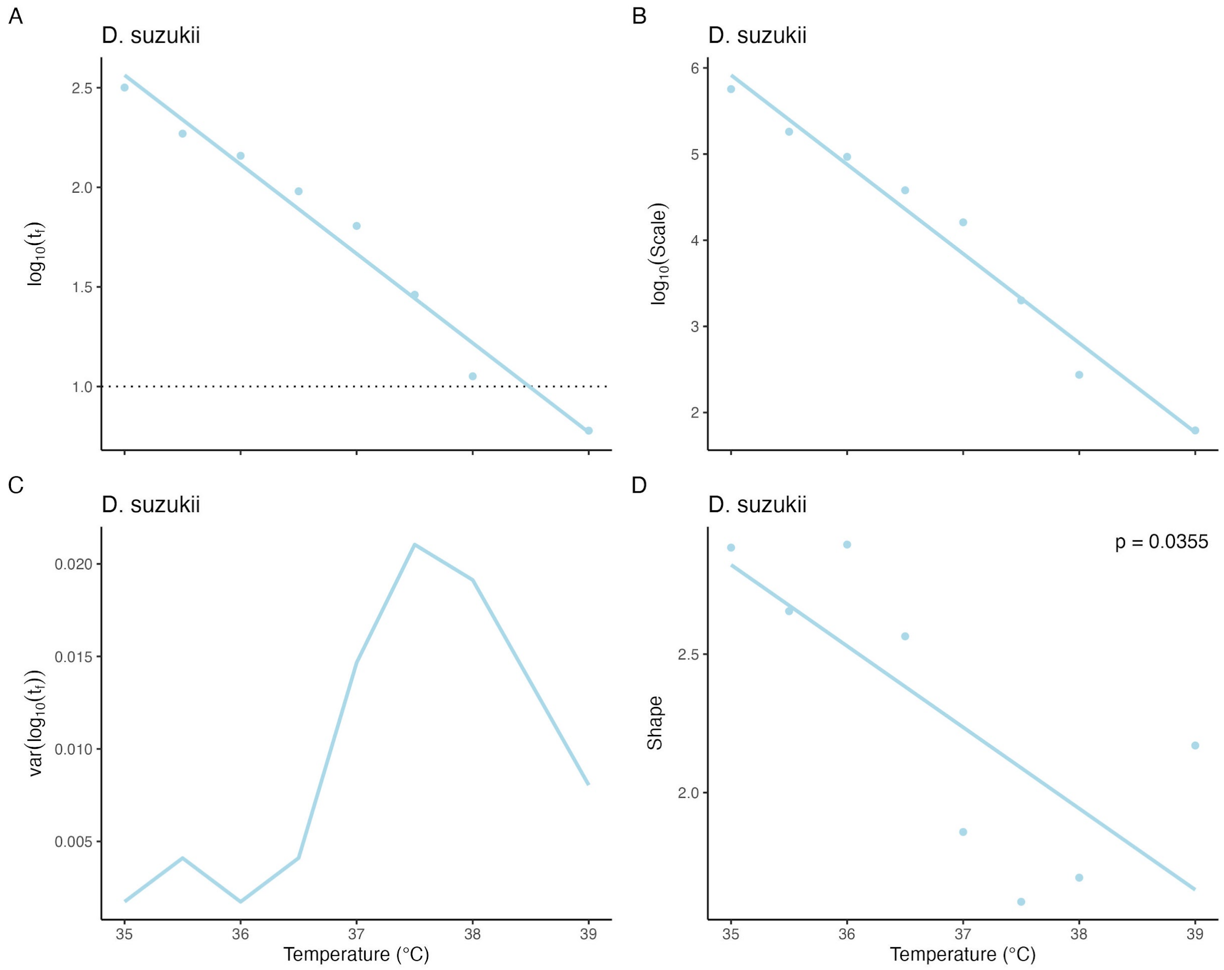
A
D. suzukii
$\log_{10}(t_f)$
2.5
2.0
1.5
1.0
B
D. suzukii
$\log_{10}$(Scale)
6
5
4
3
2
C
D. suzukii
var($\log_{10}(t_f)$)
0.020
0.015
0.010
0.005
35
36
37
38
39
Temperature (°C)
D
D. suzukii
p = 0.0355
Shape
2.5
2.0
35
36
37
38
39
Temperature (°C)

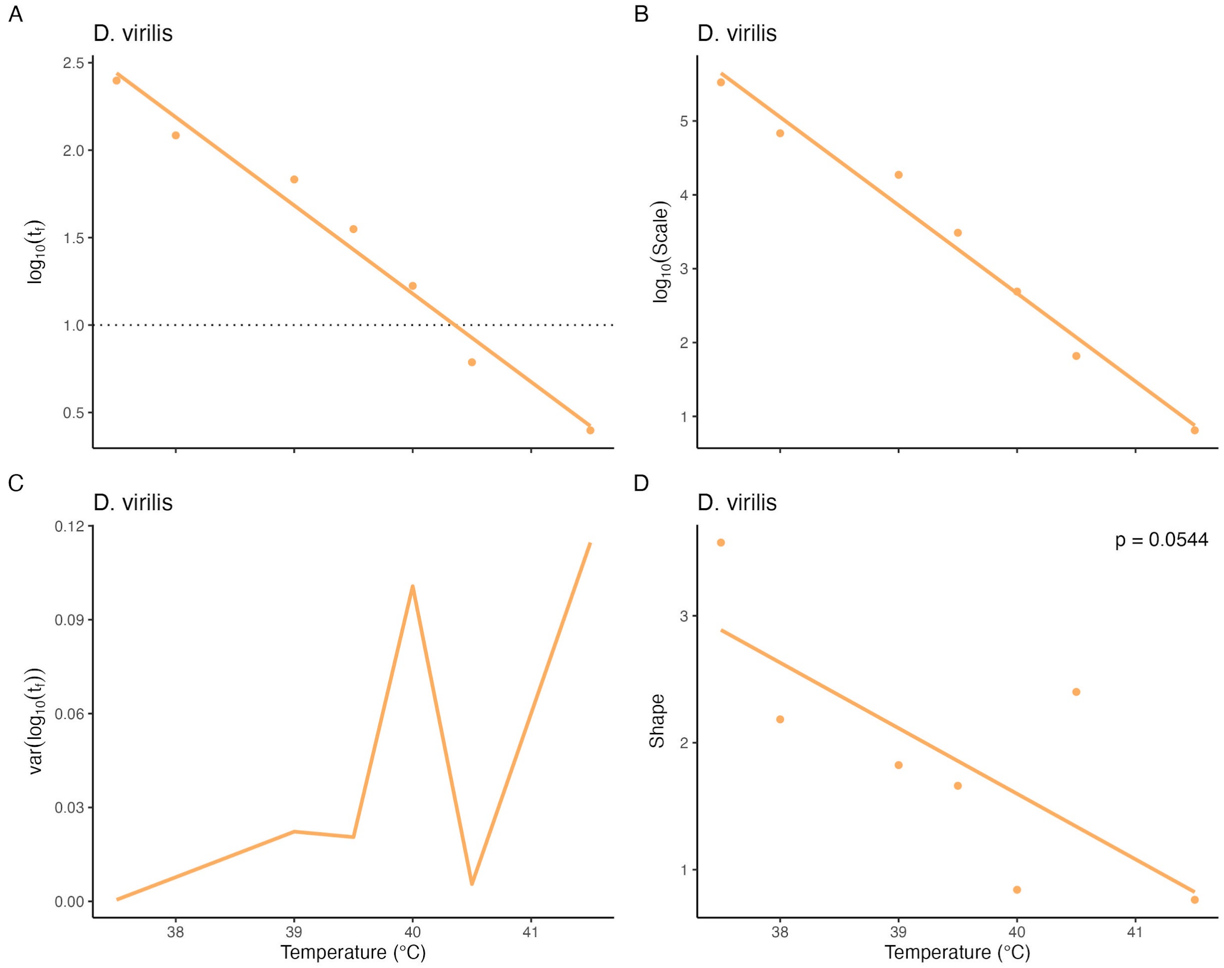
A
D. virilis
log10(tf)
2.5
2.0
1.5
1.0
0.5
B
D. virilis
log10(Scale)
5
4
3
2
1
C
D. virilis
var(log10(tf))
0.12
0.09
0.06
0.03
0.00
38
39
40
41
Temperature (°C)
D
D. virilis
p = 0.0544
Shape
3
2
1
38
39
40
41
Temperature (°C)
