# Supplementary material for: Incorporating variation in death times improves predictions of ectotherm responses to stressful temperatures
Source: PLoS Biol. 2026 May 21;24(5):e3003623. doi: 10.1371/journal.pbio.3003623 (PMC13221141; doi:10.1371/journal.pbio.3003623)
Supplement: S4 Fig — Species are those not included in Fig 3. Curves for the observed data (gray lines) and predictions based on the Rezende and colleagues (orange) and increasing variance (purple) models are given for each temperature. The data underlying this Figure can be found in https://zenodo.org/records/1937403. (PDF) [file pbio.3003623.s007.pdf]

S4 Figure. Observed and predicted failure densities and cumulative survival curves for 9 *Drosophila* species at two constant temperatures. Species are those not included in Figure 3. Curves for the observed data (grey lines) and predictions based on the Rezende *et al* (orange) and increasing variance (purple) models are given for each temperature. The data underlying this Figure can be found in https://zenodo.org/records/1937403.

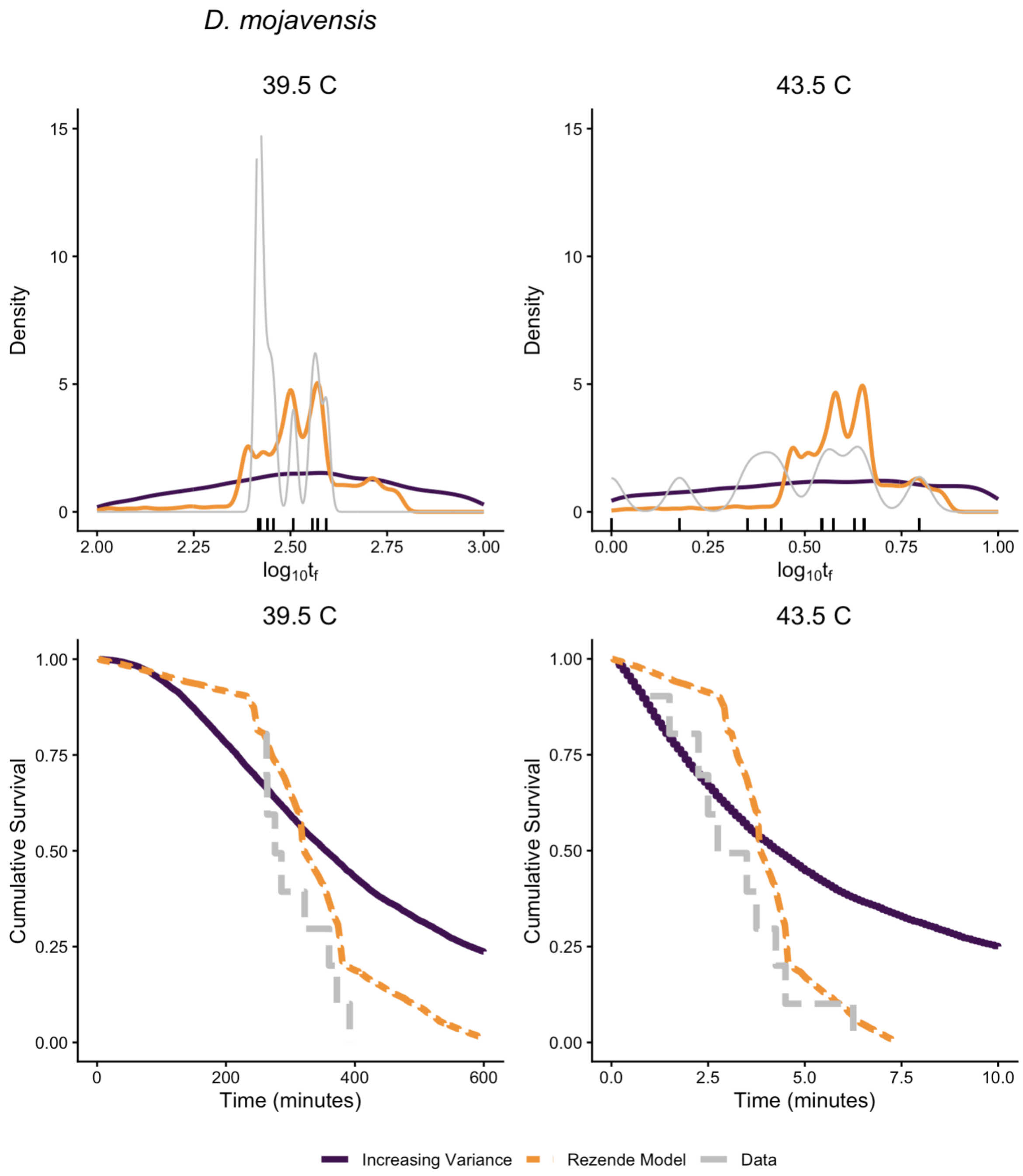

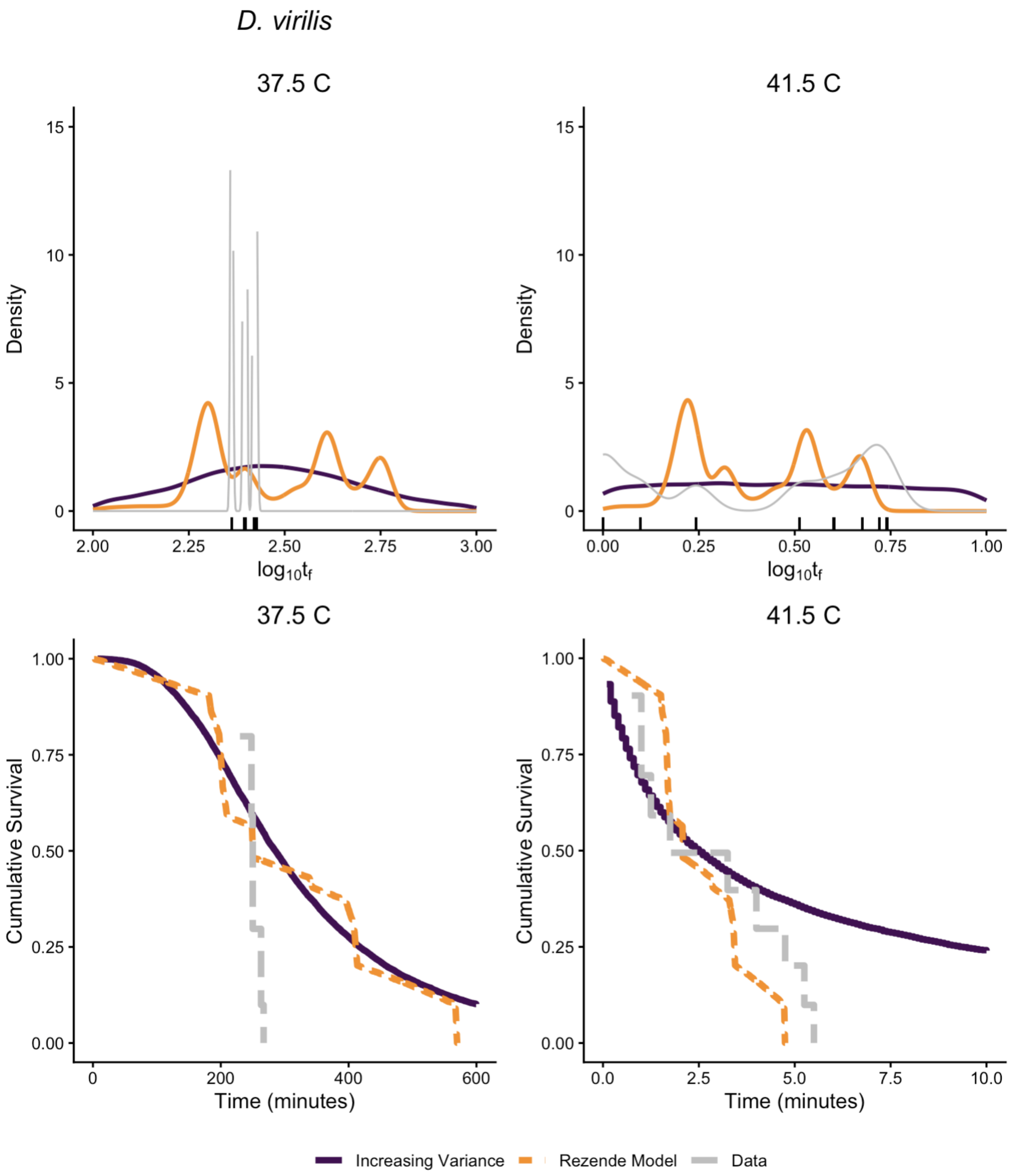

D. virilis
37.5 C
41.5 C
Density
15
10
5
0
2.00
2.25
2.50
2.75
3.00
0.00
0.25
0.50
0.75
1.00
log10tf
Cumulative Survival
1.00
0.75
0.50
0.25
0.00
0
200
400
600
0.0
2.5
5.0
7.5
10.0
Time (minutes)
Increasing Variance
Rezende Model
Data

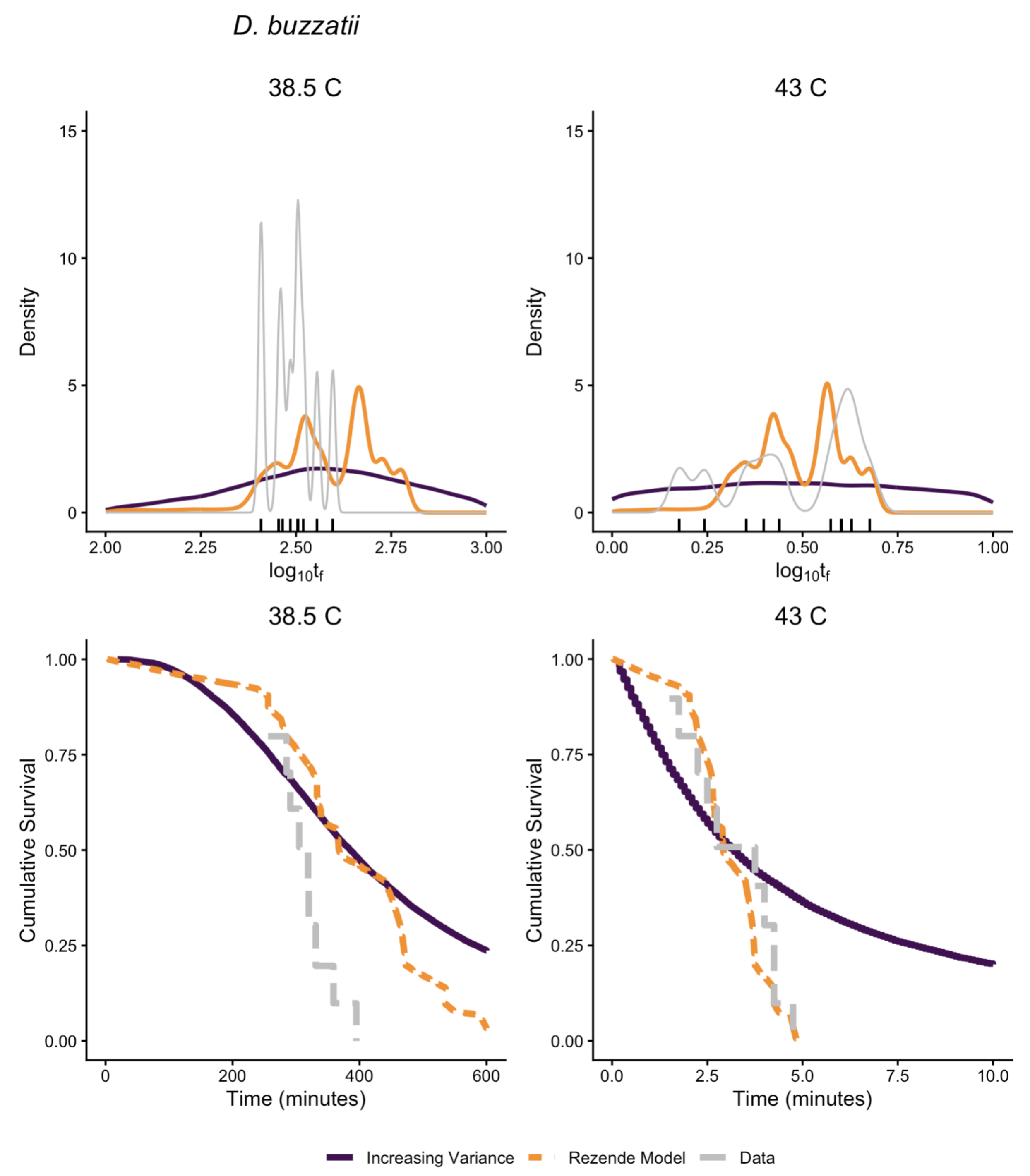

D. buzzatii
38.5 C
43 C
Density
log10tf
Cumulative Survival
Time (minutes)
Increasing Variance
Rezende Model
Data

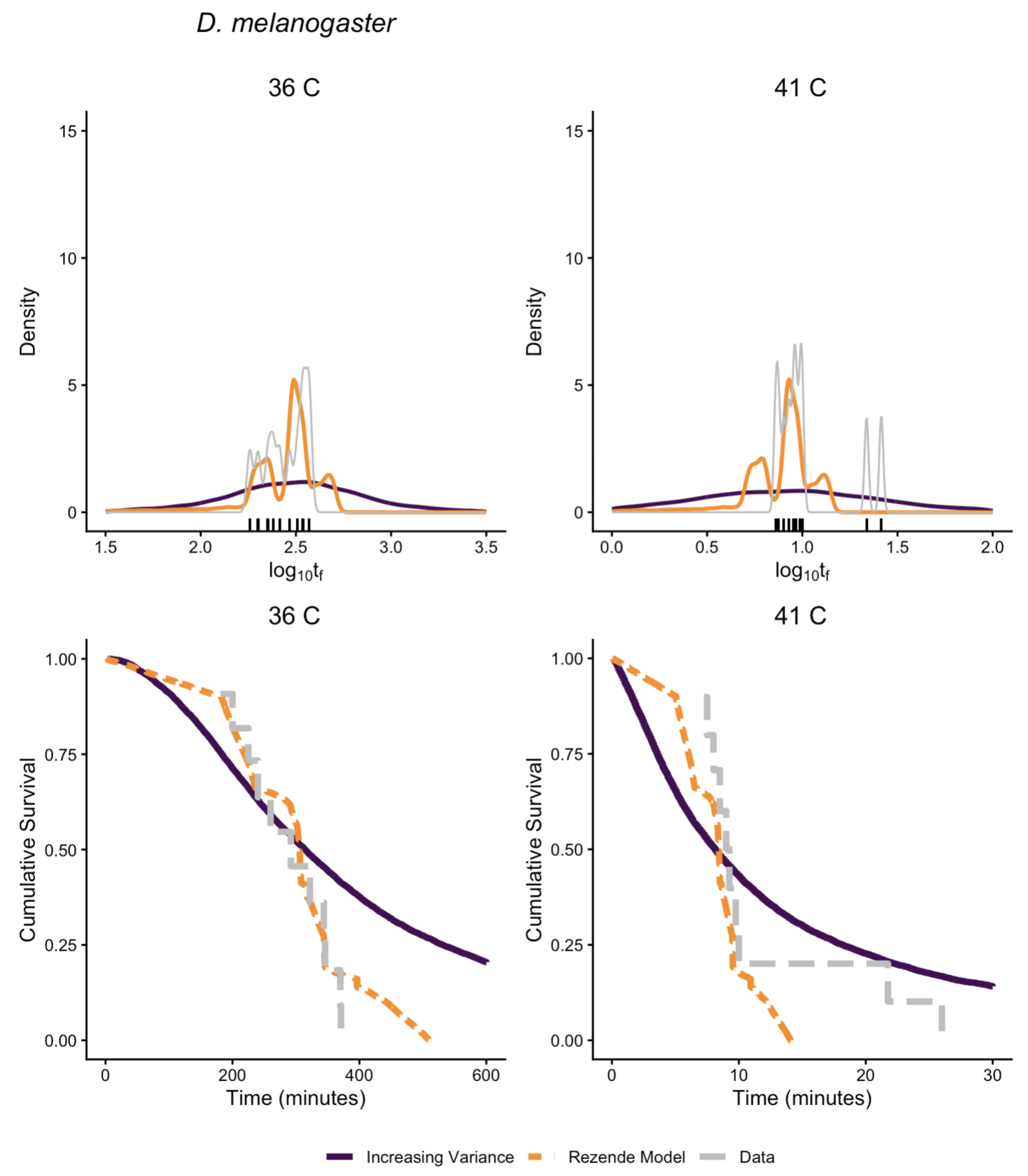

D. melanogaster
36 C
41 C
Density
$\log_{10} t_f$
Cumulative Survival
Time (minutes)
Increasing Variance
Rezende Model
Data

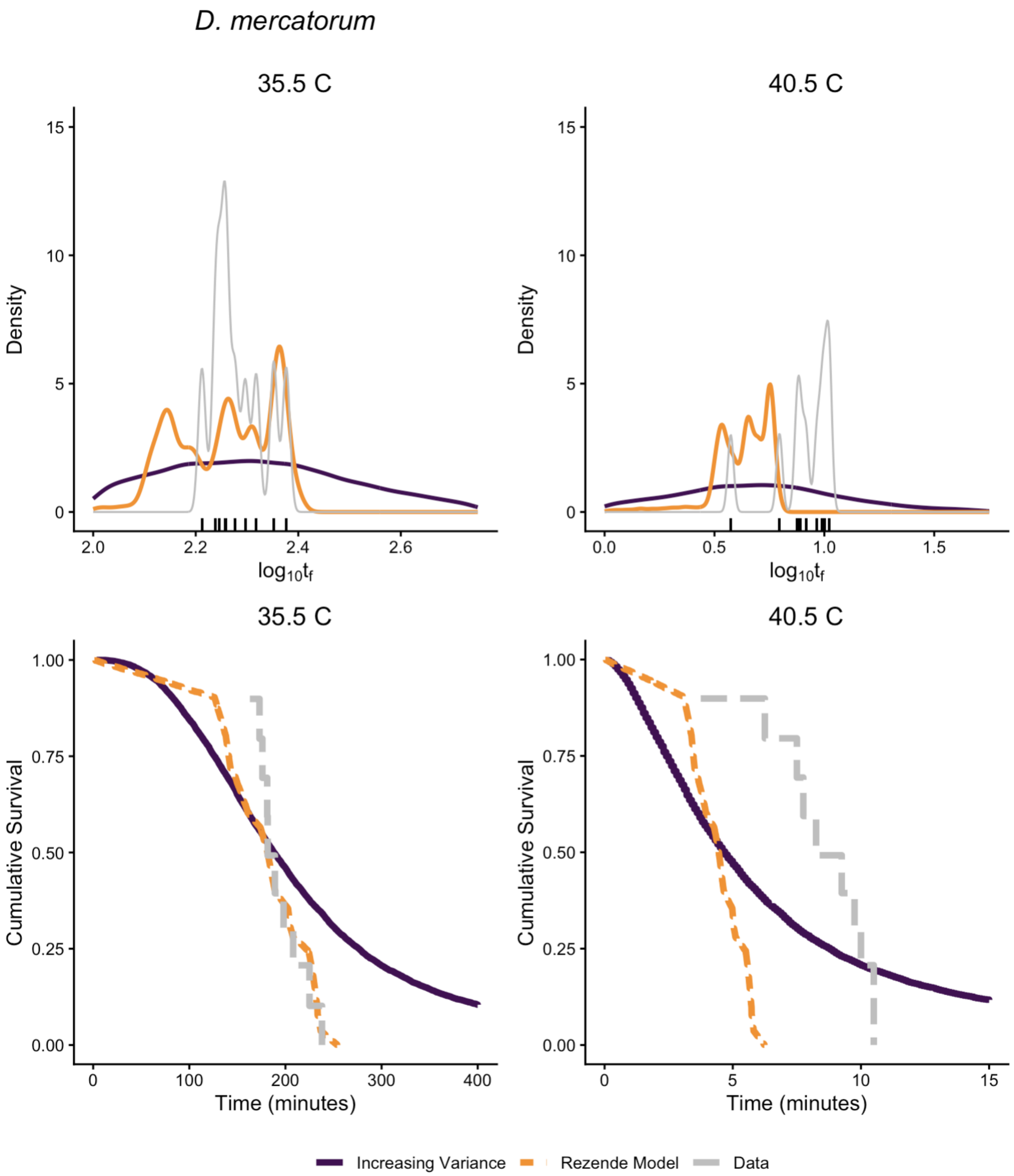

D. mercatorum
35.5 C
40.5 C
Density
log10tf
35.5 C
40.5 C
Cumulative Survival
Time (minutes)
Increasing Variance
Rezende Model
Data

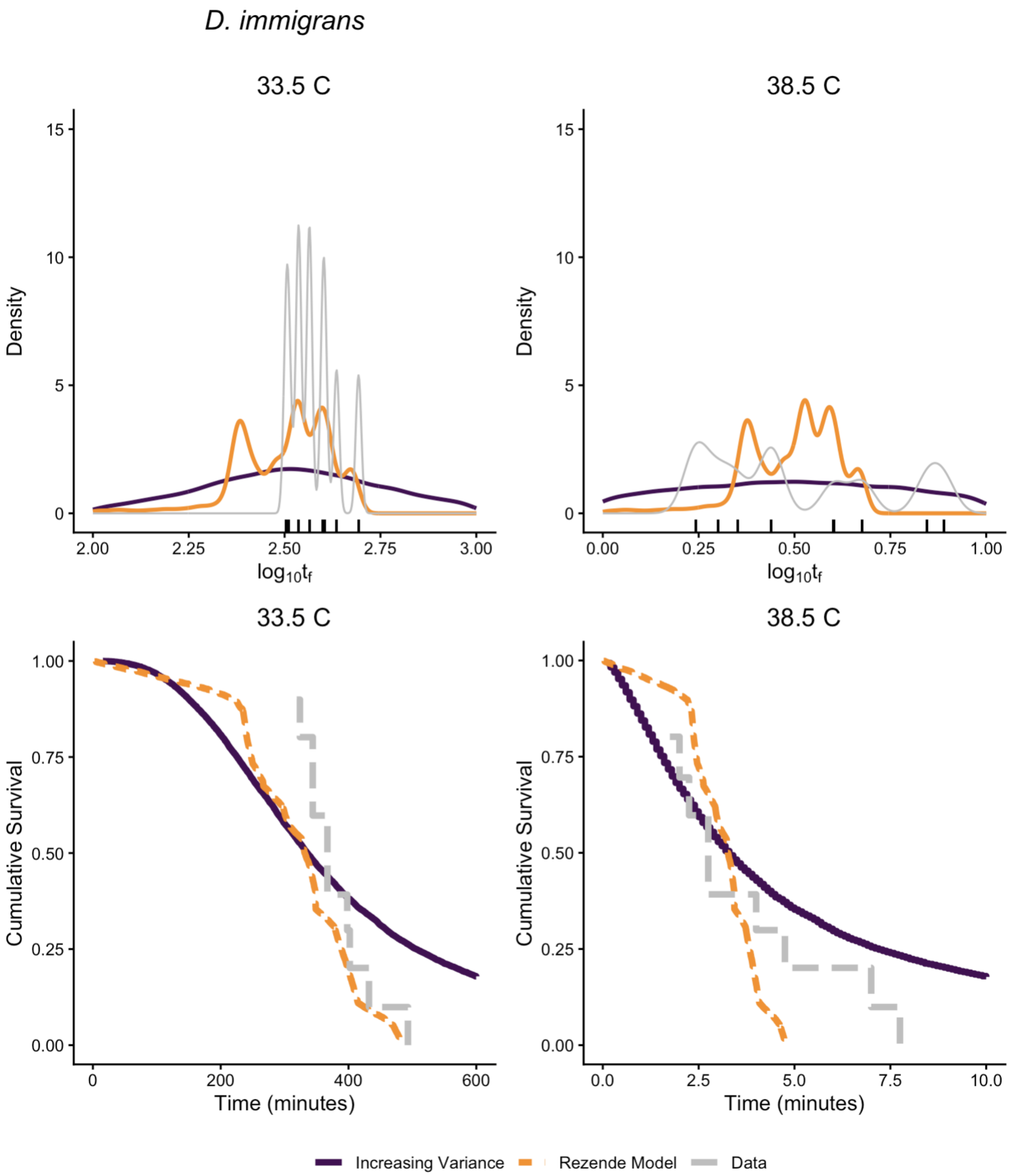

D. immigrans
33.5 C
38.5 C
Density
log10tf
Cumulative Survival
Time (minutes)
Increasing Variance
Rezende Model
Data

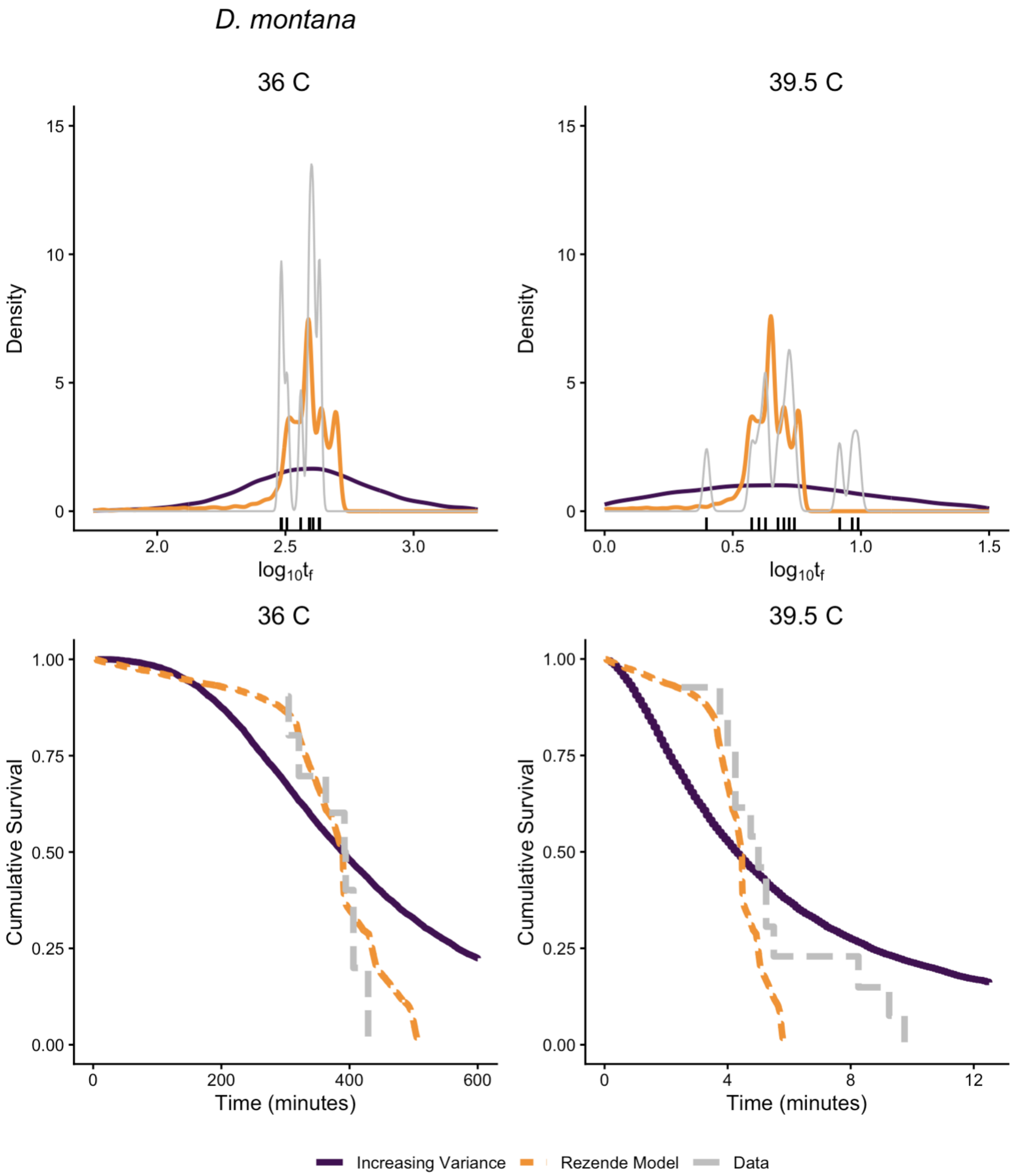

D. montana
36 C
39.5 C
Density
log10tf
Cumulative Survival
Time (minutes)
Increasing Variance
Rezende Model
Data

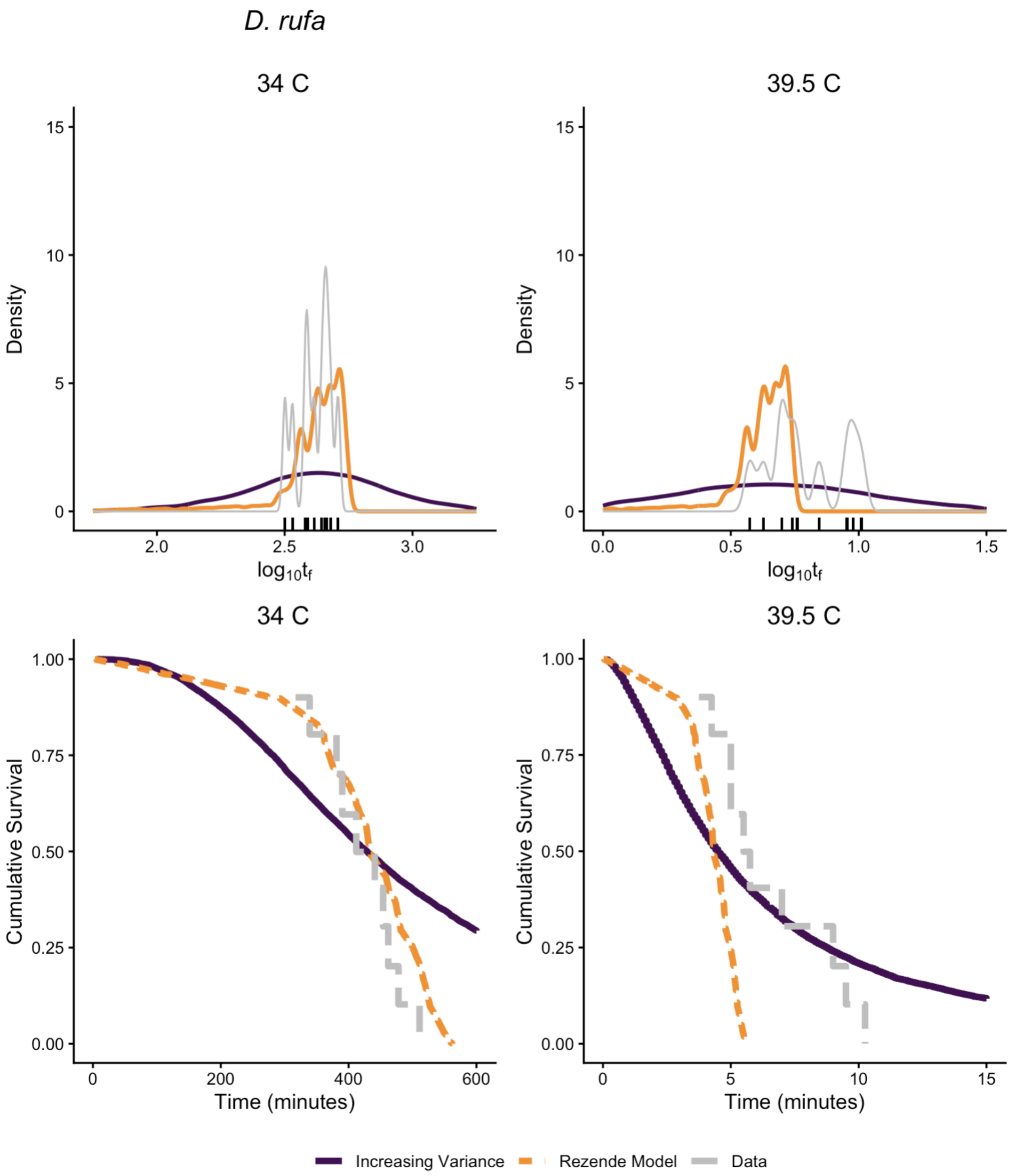
D. rufa
34 C
39.5 C
Density
15
10
5
0
2.0
2.5
3.0
0.0
0.5
1.0
1.5
log10tf
34 C
39.5 C
Cumulative Survival
1.00
0.75
0.50
0.25
0.00
0
200
400
600
5
10
15
Time (minutes)
Increasing Variance
Rezende Model
Data

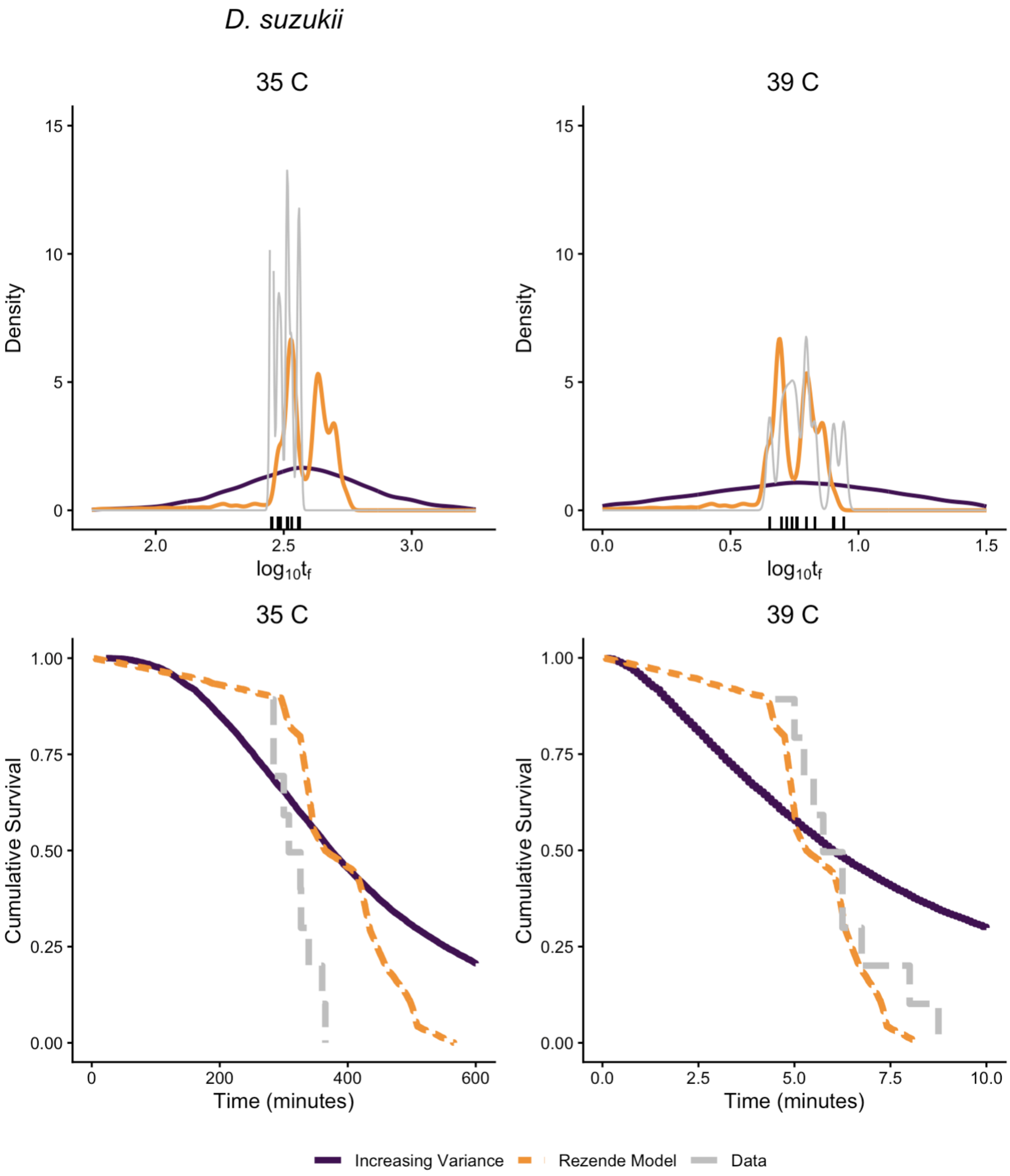

D. suzukii
35 C
39 C
Density
log10tf
Cumulative Survival
Time (minutes)
Increasing Variance
Rezende Model
Data
