# Supplementary material for: Incorporating variation in death times improves predictions of ectotherm responses to stressful temperatures
Source: PLoS Biol. 2026 May 21;24(5):e3003623. doi: 10.1371/journal.pbio.3003623 (PMC13221141; doi:10.1371/journal.pbio.3003623)

**S5 Figure. Observed vs predicted median failure times for sets of adult *D. melanogaster* in different fluctuating stressful temperature conditions.** Panel A: Females; Panel B: Males. Predictions for the log-logistic increasing variance (orange) and constant variance (gray) models. Linear regression line for each model is shown, as well as the 1:1 line (light grey line). There is little difference between these models for this population, as the *shape* vs temperature trend in the static temperature training data was minimal. Given the variation in *shape* vs temperature relationships between populations (Figure 2), we could expect these results to vary based on the population. The data underlying this Figure can be found in [https://zenodo.org/records/1937403](https://zenodo.org/records/1937403).

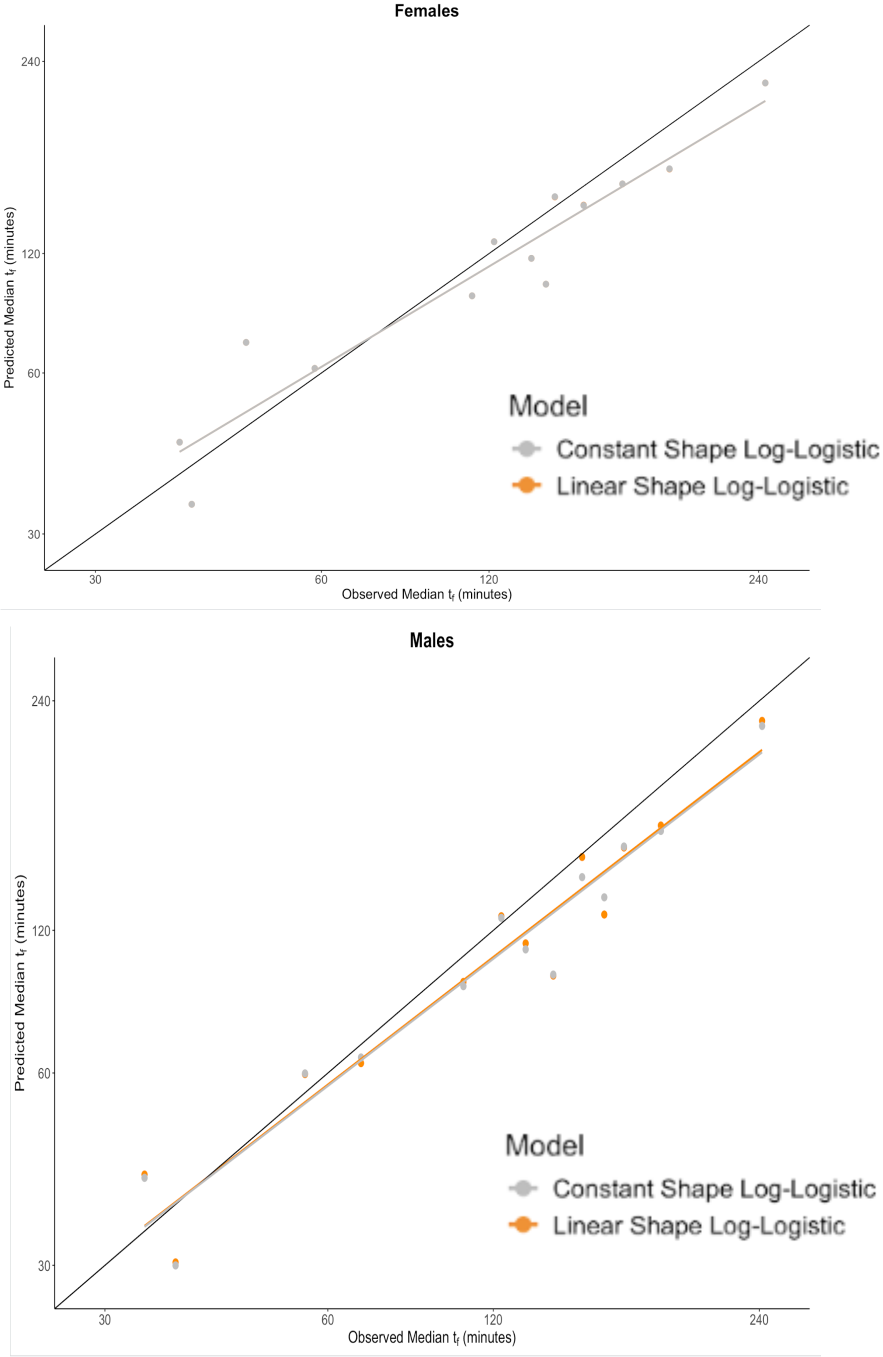

Supplement: S5 Fig — Panel A: Females; Panel B: Males. (PDF) [file pbio.3003623.s008.pdf]
