# Supplementary material for: Incorporating variation in death times improves predictions of ectotherm responses to stressful temperatures
Source: PLoS Biol. 2026 May 21;24(5):e3003623. doi: 10.1371/journal.pbio.3003623 (PMC13221141; doi:10.1371/journal.pbio.3003623)

S6 Figure. Cumulative survival curve data for 13 sets of adult *D. melanogaster* in different fluctuating stressful temperature conditions (data from (1)) with estimates from Rezende et al. and Increasing Variance models. Results are grouped by sex. The data underlying this Figure can be found in https://zenodo.org/records/1937403.

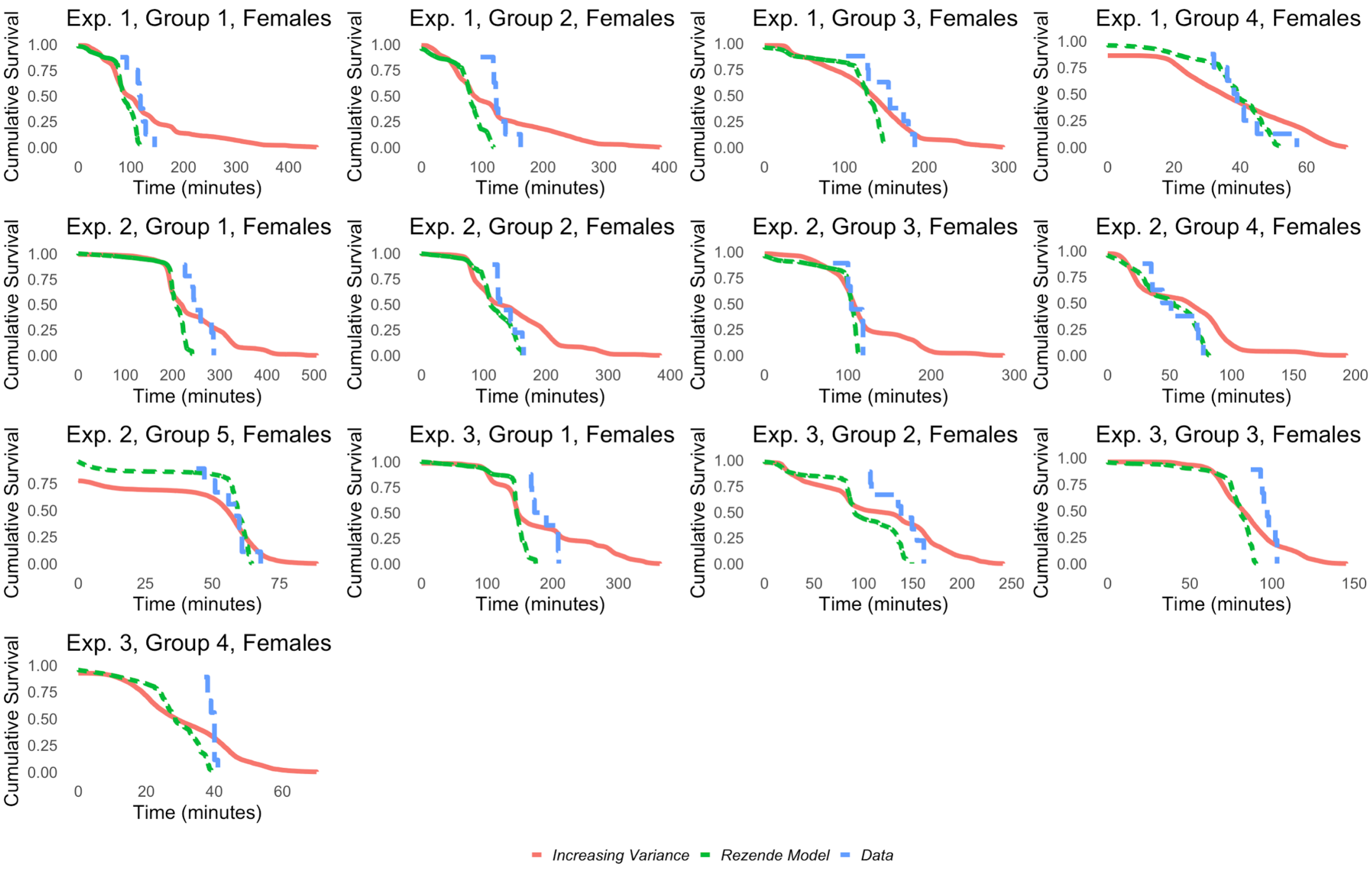

Bullard et al. PLOS Biology

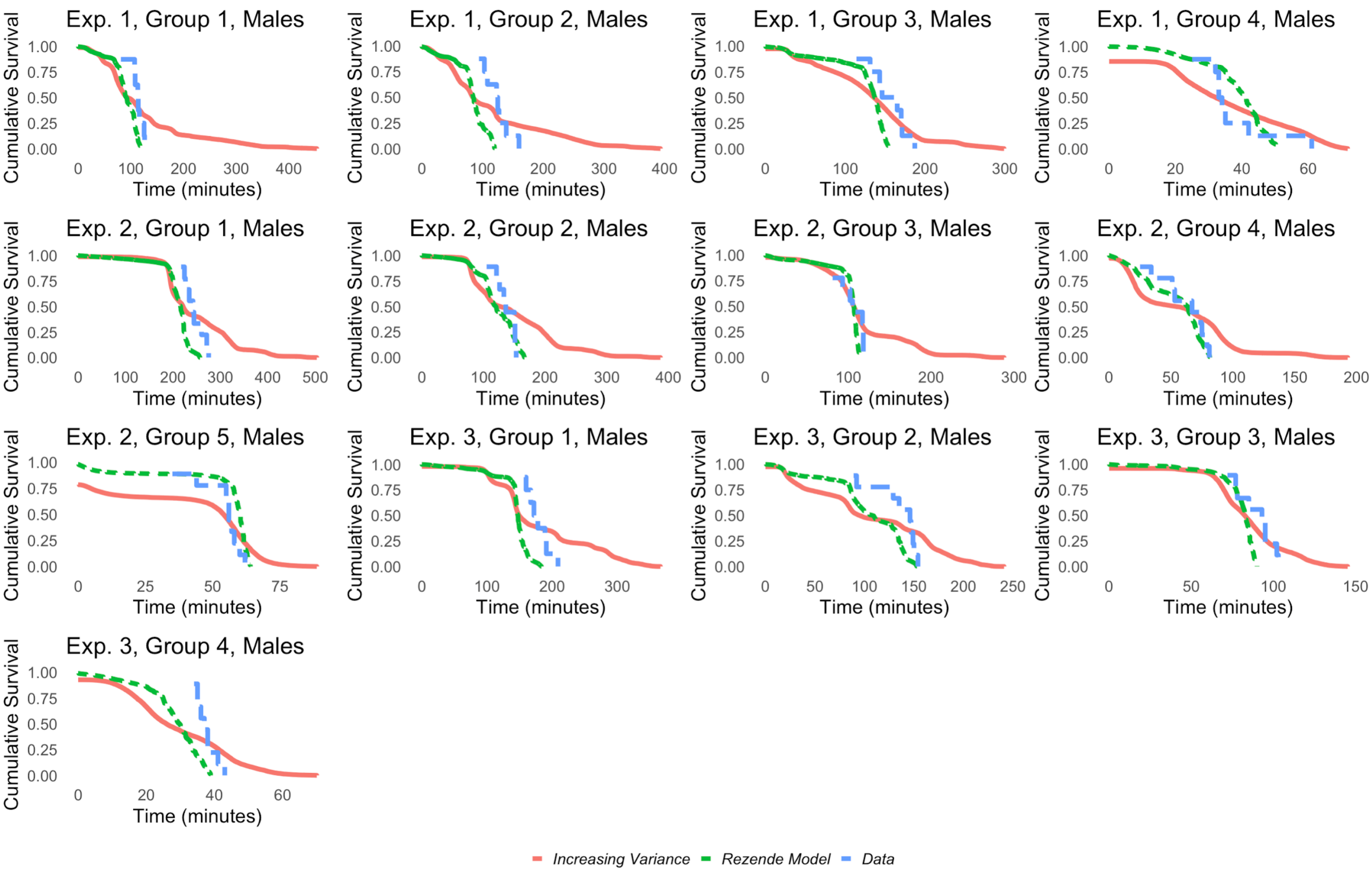

Supplement: S6 Fig — Results are grouped by sex. The data underlying this Figure can be found in https://zenodo.org/records/1937403. (PDF) [file pbio.3003623.s009.pdf]
