# Supplementary material for: Incorporating variation in death times improves predictions of ectotherm responses to stressful temperatures
Source: PLoS Biol. 2026 May 21;24(5):e3003623. doi: 10.1371/journal.pbio.3003623 (PMC13221141; doi:10.1371/journal.pbio.3003623)
Supplement: S1 Text — (PDF) [file pbio.3003623.s010.pdf]

S1 Text. Supplementary Methods

Model Comparison Details

We compared three models—Rezende et al., Jorgensen et al., and the increasing-variance model—against the fluctuating-temperature failure data from Jorgensen et al. (2021). The models differ in the type of output they produce. The deterministic Jorgensen model predicts only a median failure time. The nonparametric stochastic model of Rezende et al. outputs a nonparametric survival curve (1 – cumulative density function (CDF) of failure time). The increasing-variance model predicts a smooth, parametric survival curve.

Model performance was evaluated using two metrics: median failure time and mean log-likelihood. Median failure time was a natural choice because the deterministic model predicts only this quantity. The median-based comparison (Fig. 4A, B) is straightforward and described in the main text.

Log-likelihood analysis was more involved. Log-likelihood requires a probability density function (PDF), which none of the models provide directly. The deterministic model predicts only a median and therefore cannot be assigned a density. The two stochastic models return CDFs, which can be converted to PDFs. Because these CDFs have no closed-form expression, PDFs were estimated via simulation.

Specifically, given a survival function $S(t)$ predicted under fluctuating temperature conditions $T(t)$, and empirical failure times $x_1, x_2, \ldots, x_n$:

1. Draw 10,000 samples $u_i \sim U(0,1)$
2. Transform them via $t_i = S^{-1}(u_i)$ to obtain samples from the implied PDF
3. Estimate the PDF $f(t)$ using kernel density estimation
4. Compute log-likelihoods of the observed data under $f(t)$
5. Average log-likelihoods to account for unequal sample sizes
6. Repeat for each fluctuating-temperature trial and both stochastic models

Variance Assumption Explanation

Existing Thermal Death Time models assume the following:
Where $t_f(T)$ is the time of death at temperature T, for all $T > T_C \rightarrow \text{var}(\log(t_f)) = c$.
This relationship comes from the constant variance assumption of the linear TDT relationship: $log(t_f) \sim norm(\mu = \beta_0 + \beta_1 * T, \sigma^2 = c^2)$, or from the constant variance assumption of the models from Rezende et al. (2020). But formally, it implies that the variance-stabilizing transformation for our regression is $\log(t_f)$:

$var(log(t_f(T))) = c \; for \; all \; T > T_C,$
Where T_C is the “critical threshold between stressful and permissive temperatures.
For the Log-Logistic regression, it can be shown that log is the variance-stabilizing function only when shape is constant across stressful temperatures:

$$var(ln(t_f(T))) = \frac{\pi^2}{3k^2} = c_1 \; iff \; k(T) = c_2 \; for \; all \; T > T_C$$

Note this statement holds regardless of the relationship between α and temperature. A change of logarithmic base just introduces a constant scalar to the equation:

$$var(log_{10}(t_f(T))) = \frac{\pi^2}{ln(10)^2 * 3k^2}$$

We exchange the natural log for log base 10 for interpretability and consistency with previous literature. Therefore, in assuming that variance is stabilized by the log function, one also implicitly assumes a constant Log-Linear shape *k*.
